# Acute-phase proteins during inflammatory reaction by bacterial infection: Fish-model

**DOI:** 10.1038/s41598-019-41312-z

**Published:** 2019-03-18

**Authors:** Ives Charlie-Silva, Andre Klein, Juliana M. M. Gomes, Ed J. R. Prado, Alessandra C. Moraes, Silas F. Eto, Dayanne C. Fernandes, José J. Fagliari, José D. Corrêa Junior, Carla Lima, Mônica Lopes-Ferreira, Katia Conceição, Wilson G. Manrique, Marco A. A. Belo

**Affiliations:** 1 Department of Preventive Veterinary Medicine, São Paulo State University (UNESP), Jaboticabal, SP Brazil; 2 Department of Pharmacology and Morphology, ICB-UFMG, Belo Horizonte, MG Brazil; 3 Immunoregulation Unit of the Special Laboratory of Applied Toxicology (CEPID/FAPESP), Butantan Institute, São Paulo, Brazil; 4 Laboratório de Bioquímica de Peptídeos, UNIFESP, São José dos Campos, SP Brazil; 5 Veterinary College, Federal University of Rondonia, Rolim de Moura, RO Brazil; 6 Laboratory of Animal Pharmacology and Toxicology, Brasil University, Descalvado, SP Brazil

## Abstract

Acute-phase protein (APPs) serum levels have been studied in many human diseases, and their components contribute to host defense during the evolution of infectious diseases by acting as part of the innate immune system. Based on the importance of establishing new experimental models, the present investigation evaluated the modulation of APPs following inflammatory stimulus by the inoculation of *Aeromonas hydrophila* in tilapias. Fish were sampled 6 and 24 hours post-infection. Tilapias presented increase of positive APPs such as ceruloplasmin, haptoglobin, alpha-2-macroglobulin and complement C3, as well as decrease of negative APPs such as albumin and transferrin. The protein response of tilapias during the course of bacterial infection showed correlation with the kinetics of cellular accumulation in the inflamed focus with significant increase of granulocytes, thrombocytes, lymphocytes and macrophages. However, granulocytes were the predominant cells, associated with increment in the reactive oxygen species (ROS) production. Showing responses similar to those observed in humans, the modulation of APPs and the kinetics of cellular accumulation in the exudate demonstrate the feasibility of this alternative experimental model for advances and studies to understand changes in pathophysiological mechanisms of acute inflammatory reaction due to bacterial infection.

## Introduction

Inflammation is a body defense mechanism in response to harmful stimuli, and is an essential component of normal tissue homeostasis^1^. After tissue injury, the inflammatory cells (neutrophils and monocytes/macrophages) secrete cytokines into the bloodstream, stimulating hepatocytes to produce proteins, which are directly involved in the body’s defense mechanisms. These are known as acute phase proteins (APPs), and their plasma levels can be modulated rapidly at the onset of inflammation^2^.

To elucidate and understand the dynamics of the pathophysiological events during inflammatory reactions, variations of APPs serum levels have been studied in many diseases, such as type 2 diabetes^3^, Alzheimer’s disease^4^, the risk of Parkinson’s disease^5^, the evolution of cancers^6^ and in cases of bacterial infections^7^.

There is increasing evidence that the components of APP responses contribute to host defense during the evolution of infectious diseases by acting as part of the innate immune system. An APP whose plasma concentration increases by at least 25% during inflammatory disorders has been called “positive” APP or “negative” AAP when they decrease in similar proportions^8^. The increment of “positive” APPs, such as C-reactive protein, complement system factors, ferritin, ceruloplasmin, haptoglobin and alpha-2-macroglobulin, among others, assists in the destruction or inhibition of microorganisms’ growth, while “negative” APPs such as albumin, transferrin, transthyretin, antithrombin and transcortin decrease during inflammation^8^.

Teleostean fish have demonstrated several advantages over other animal species, and can be an alternative to the use of experimental models with rodents. As well as, they can provide complementary information when used as a model for disease studies, prospecting of new drugs, among others^9^. For decades, classical experimental models with mammals have been used to study the evolution of inflammatory response in either acute or chronic phase. The aerocystitis model, which mimics the pleurisy technique in rodents, proved to be extremely effective for the study of acute inflammation in teleostean fish^10–13^. The swim bladder is a delimited cavity organ with terminal circulation and allows easy access for the inoculation of phlogogens and the collection of exudates for the evaluation of cellular components and fluids accumulated in the inflammatory focus^10–13^.

*A. hydrophila* is becoming food and waterborne pathogens of increasing importance, and it has been associated with several food-borne outbreaks^14,15^. In humans, this bacteria cause gastroenteritis, peritonitis, meningitis and disseminated infections in immunocompromised hosts^16^. In 2001, the United States Environmental Protection Agency validated the “Method 1605” for detection and enumeration of *Aeromonas* in drinking water system^17^.

Based on the importance of establishing new experimental models and the advantages of using teleostean fish to study the pathophysiology of the inflammatory reaction, considering that the main endogenous glucocorticoid of fish is cortisol, similar to humans^18,19^, the present investigation evaluated the modulation of APPs following inflammatory stimulus by the inoculation of *Aeromonas hydrophila* into the swim bladder of tilapias, correlating such levels to the cellular components present in the fish blood and exudates during the evolution of acute inflammation (aerocystitis).

## Results

### Protein identification of variable protein bands

**Figure 1:**
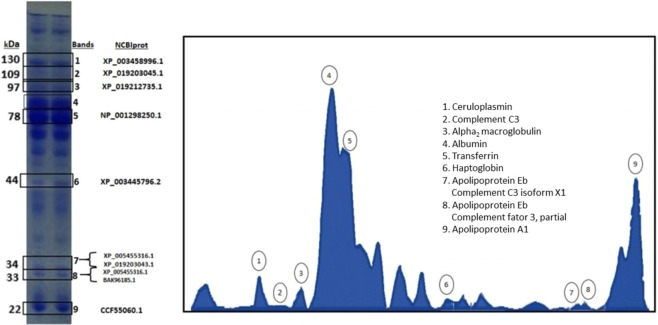
(**A**) APPs (ceruloplasmin, complement C3, α2 macroglobulin, albumin, transferrin, haptoglobin, apolipoprotein A1, complement C3 isoform X1, complement factor 3, and apolipotrotein Eb) determined by SDS polyacrylamide gel 10%, both columns obtained from control fish (*Oreochromis niloticus*). (**B**) Densitometry tracing.

Electrophoretic traces of 30 protein fractions were found by computerized densitometry whose molecular weights ranged from 22 to 200 kDa. As an attempt to characterize the acute phase proteins from the plasma of *Oreochromis niloticus*, and according to the experience of our research group on inflammation, we selected 9 differential bands (Fig. 1A,B) to in gel trypsin digestion coupled to mass spectrometric analysis resulting in the identification of proteins present in the bands. By LC-MS/MS analysis, proteins of the main acute phase proteins including ceruloplasmin, complement C3, α_2_ macroglobulin, albumin, transferrin, haptoglobin, apolipoprotein A1, complement C3 isoform X1, complement factor 3, and apolipoprotein Eb, as well as the less abundant components were identified in the bands of variable intensity at the molecular mass range of 10–122 kDa, confirming the significant variability observed on the *O. niloticus* specimens SDS-PAGE profiles (Fig. 1). Notably, some bands of molecular mass ~34 kDa and lower contained apolipoprotein Eb, complement C3 isoform X1 and, complement factor 3, indicating that the latter might be derived from protein autolysis (bands 7 and 8), due to characteristics of the complement C3 (and also C4) proteins^20,21^. Overall, the majority of the identified bands contained more than one type of protein indicating the co-migration of polypeptide chains of different proteins yet with similar molecular masses.

**Figure 2:**
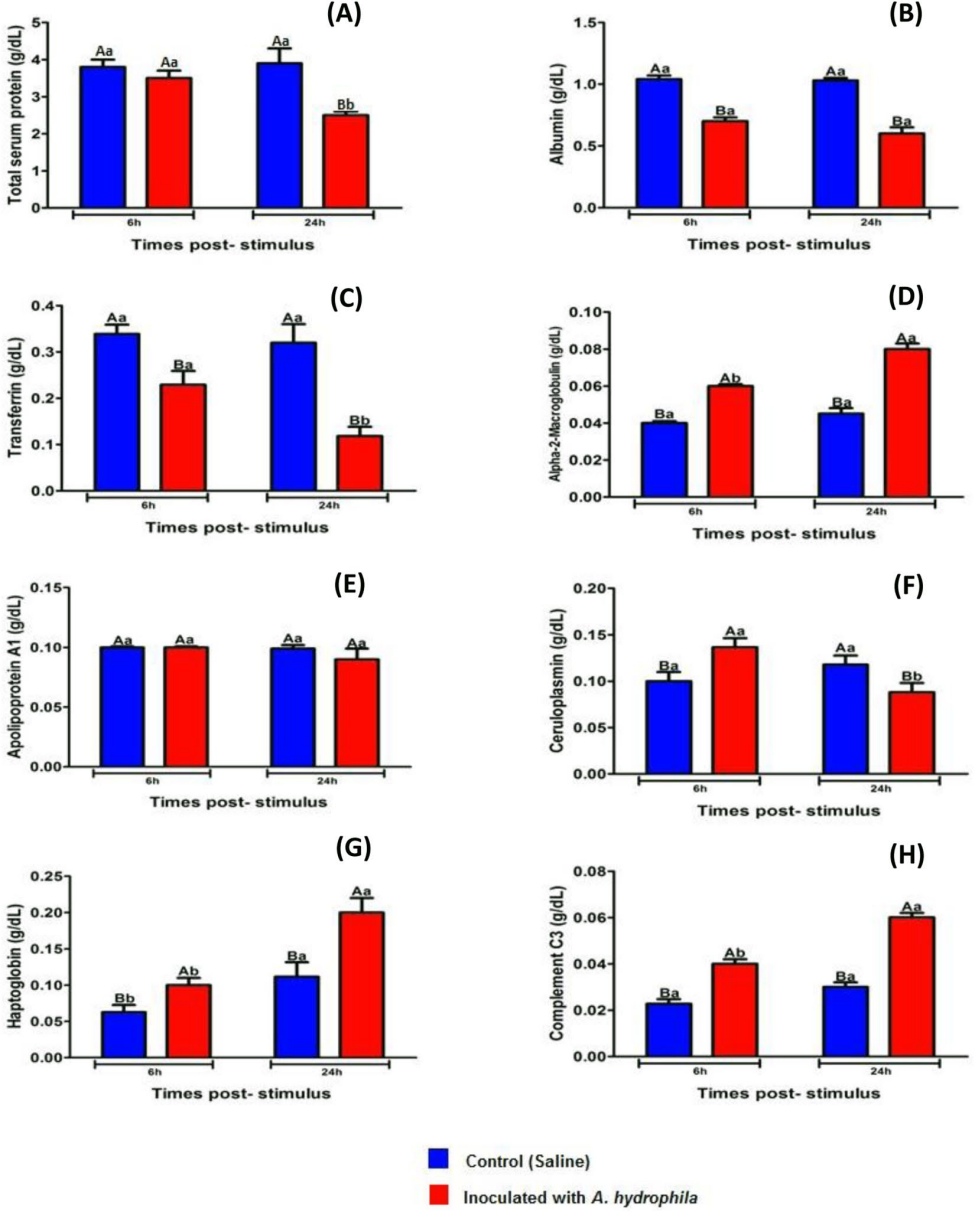
Mean values (n = 10) and ANOVA observed for APPs [total protein (**A**), albumin (**B**), transferrin (**C**), α2 macroglobulin (**D**), apolipoprotein A1 (**E**), ceruloplasmin **(F**), haptoglobin (**G**) and complement C3 (**H**)] during acute inflammatory response in tilapias 6 and 24 HPI. Means followed by the same letter do not differ by the Tukey test (P < 0,05). The variance analysis is represented by capital letters to compare the different treatments within each experimental period, lowercase letters to compare the evolution of each treatment in the different experimental periods. Different letters indicate significant difference (p < 0.05).

Fish inoculated with *A. hydrophila* showed a significant decrease (P < 0.05) in the amount of albumin and transferrin when compared to control animals 6 and 24 HPI, as well as decrease in total protein 24 HPI (Fig. 2). The levels of haptoglobin, α2 macroglobulin and complement C3 increased significantly (P < 0.05) in tilapia blood with infectious aerocystitis when compared to control fish 6 and 24 HPI (Fig. 2), and regarding to the inflammatory reaction evolution, these three proteins showed a significant increase (P < 0.05) between the periods of 6 and 24 HPI (Fig. 2).

Infected tilapias presented significant variations (P < 0.05) in ceruloplasmin blood levels with an increase 6 HPI, followed by a decrease with 24 HPI in comparison to the values observed in the control animals (Fig. 2). There were no significant changes in serum levels of apolipoprotein A1 in tilapia submitted to different treatments, as well as in the evolution of both treatments over time (Fig. 2).

### Identification and characterization of acute phase proteins

**Figure 3:**
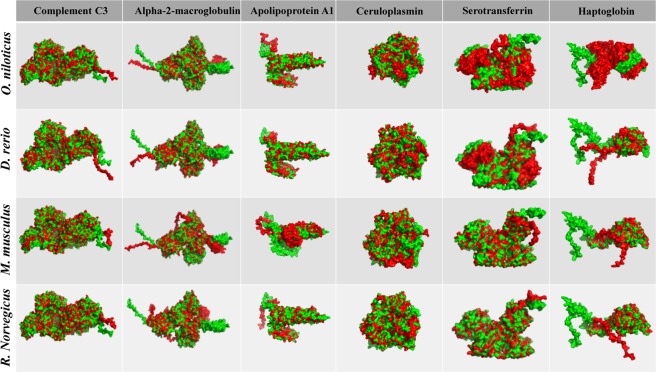
Structural similarities of tilapia serum proteins. The PDB files were generated by Raptor X2 and the structural overlay analyzed in the iPDA1. Green represents the molecule of *Homo sapiens* and red compared species (*Oreochromis niloticus*; *Danio rerio*; *Mus musculus* and *Rattus Norvegicus*).

Protein structural similarities of *Oreochromis niloticus* and *Danio rerio* were compared to homologous proteins of *Homo sapiens* and the percentage of similarity observed in the alignment converges and bases the hypothesis of equivalence among them (Fig. 3 and Table 1). In addition, the comparative study between *Mus musculus* and *Rattus Norvegicus* with *H. sapiens* homologous proteins (two established and validated experimental models) serves as a parameter of similarity, demonstrating the biological proximity between the two aquatic models with humans (Fig. 3 and Table 1**)**.

**Table 1:** Comparative alignment between the acute phase proteins of human serum with fish and rodents’ homologous proteins.

Species	Similarity (%)^a^					
	Complement C3	Alpha-2-macroglobulin	Apolipoprotein A1	Ceruloplasmin	Serotransferrin	Haptoglobin
***O. niloticus/H. sapiwens***	57.8% (I3KHS6*/*P01024)^b^	54.5% (I3K5B8*/*P01023)	42.7% (I7J7R5*/*P02647)	67.0% (I3JNN4*/*P00450)	49.8% (I3J919*/*P02787)	53.5% (I3J4H8*/*P00738)
***D. rerio/H. sapiens***	62.5% (F1QYN0*/*P01024)	56.3% (A0JMP8*/*P01023)	48.9% (O42363*/*P02647)	71.2% (Q6P3G1*/*P00450)	58.5% (B8JL43*/*P02787)	50.2% (F8W5P2*/*P00738)
***M. musculus/H. sapiens***	87.8% (P01027*/*P01024)	83.7% (Q6GQT1*/*P01023)	80.5% (Q00623*/*P02647)	90.7% (Q61147*/*P00450)	82.9% (Q921I1*/*P02787)	76.4% (Q61646*/*P00738)
***R. norvegicus/H. sapiens***	88.4% (P01026*/*P01024)	84.8% (P06238*/*P01023)	76.2% (P04639*/*P02647)	89.6% (P13635*/*P00450)	83.0% (P12346*/*P02787)	76.6% (P06866/00738)

^a^EMBOSS Water was used for pairing. Horizontal line of the table shows species compared percentage (%) of similarity and respective access number. ^b^UNIPROT.

### Blood cells and Oxidative Burst

**Figure 4:**
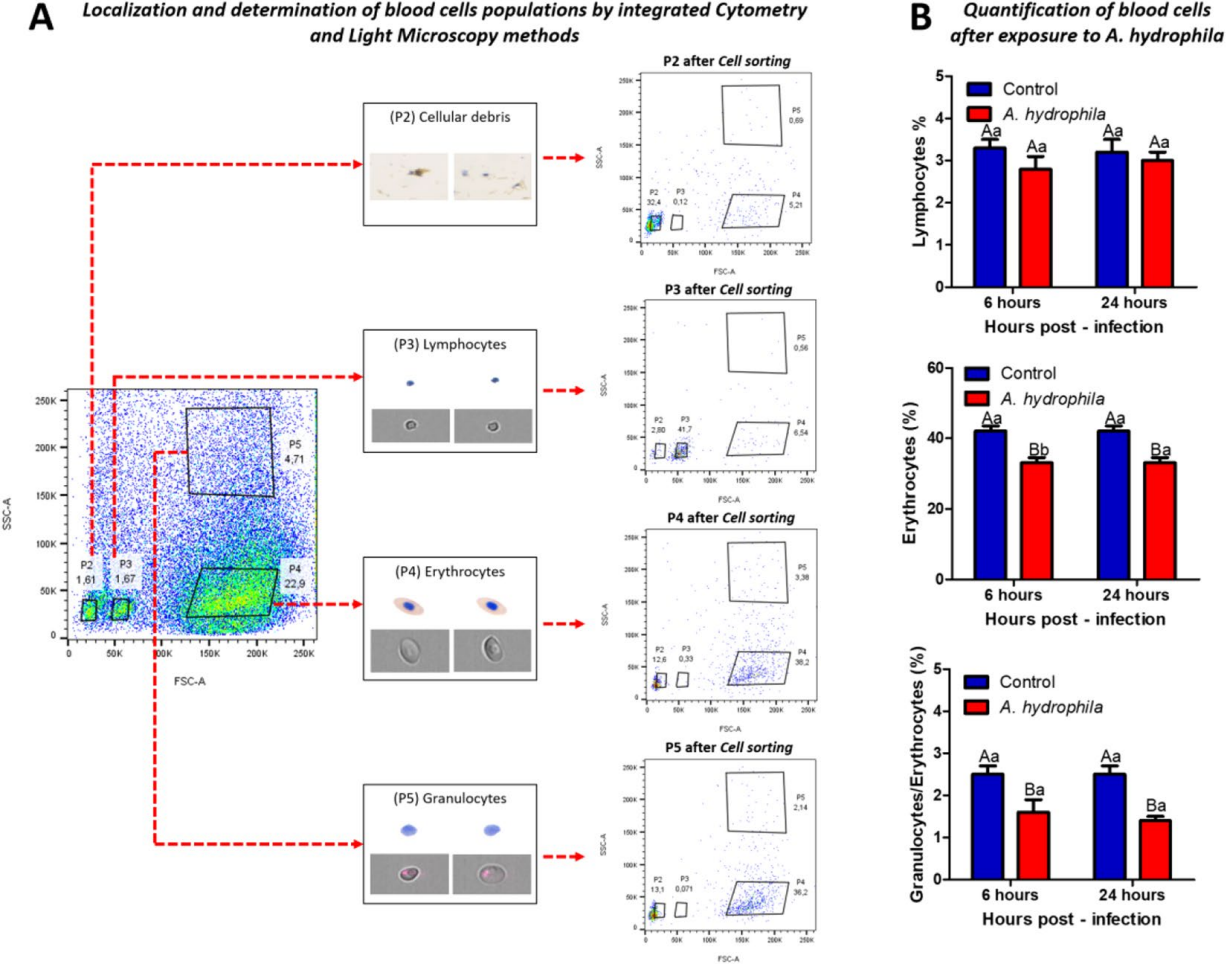
Blood cells of *O. niloticus* separated by FACSAria: (**A**) Schematic image of the place where the caudal puncture was made. The flow cytometry plot of total blood represents the cell profile of tilapia blood before separation by Cell sorting cytometer. The populations P2, P3, P4 and P5 were analyzed again after cell sorting in order to confirm their positions. Images acquisition by ImagemStream and light microscopy have identified the cell types of each population. P2 - cellular debris; P3 - Portion rich in lymphocytes; P4 - portion rich in erythrocytes and P5 - portion rich in granulocytes/erythrocytes. (**B**) Quantification of different blood cells after exposure to *A. hydrophila*, and mean values (n = 10) followed by the same letter do not differ by the Tukey test (P < 0,05). The variance analysis is represented by capital letters to compare the different treatments within each experimental period, lowercase letters to compare the evolution of each treatment in the different experimental periods. Different letters indicate significant difference (p < 0.05).

Analysis of several distinct populations of blood was resolved by light-scatter characteristics after rounds of cell sorting. First, it was to perform a triage of total blood cells removed by caudal puncture (Fig. 4A). Four populations were detected and it were called P2, P3, P4 and P5 (Fig. 4A) and confirmed after sorting and morphologically. Cells of each population were acquired and photographed by the cytometer ImageStream (gray background images) and by Olympus BX41 light microscope with a Q-color digital camera attached. These images showed the presence of cellular debris in P2, cells with typical morphology of lymphocytes in P3 and a mixture of erythrocytes and granulocytes in P4 and P5.

**Figure 5:**
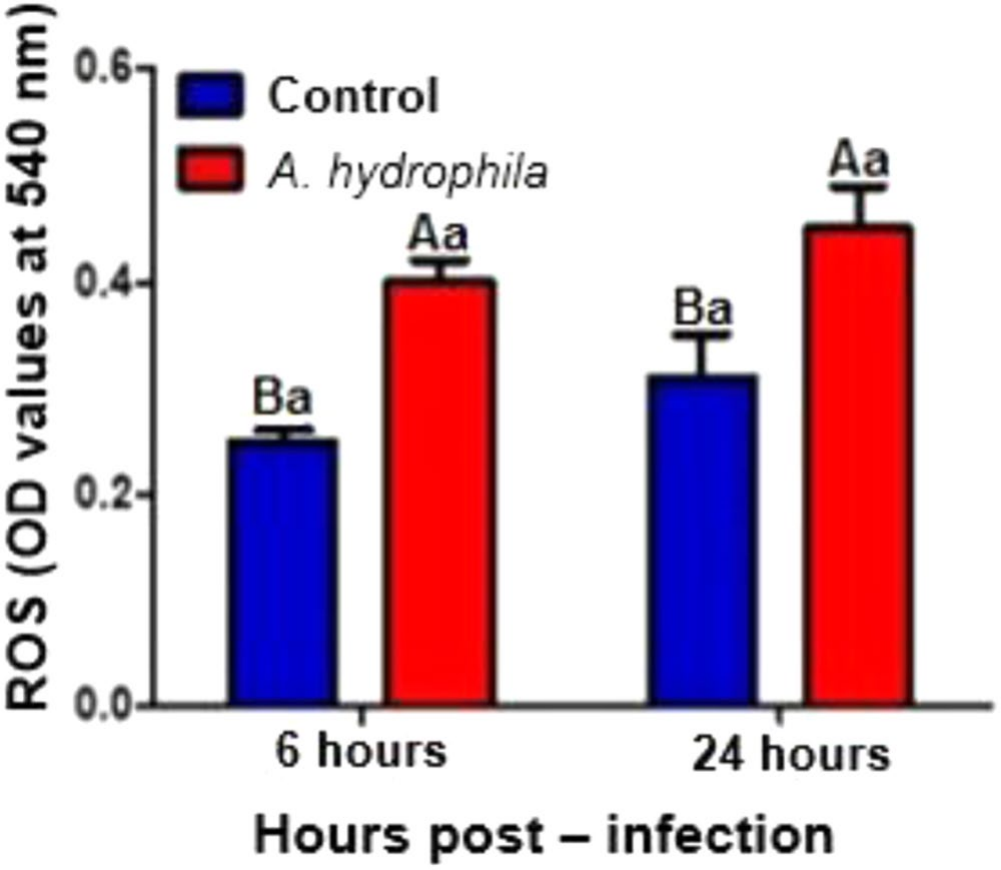
NBT assay to determinate ROS production, and mean values (n = 10) followed by the same letter do not differ by the Tukey test (P < 0,05). The variance analysis is represented by capital letters to compare the different treatments within each experimental period, lowercase letters to compare the evolution of each treatment in the different experimental periods. Different letters indicate significant difference (p < 0.05).

The animals inoculated with *A. hydrophila* showed a significant decrease (p < 0.05) in erythrocyte and granulocyte counts 6 and 24 HPI when compared to animals inoculated with saline (Fig. 4B). No difference was observed in the number of circulating lymphocytes 6 and 24 HPI (Fig. 4B). These results observed in the hematological study demonstrated a strong correlation with the findings observed in the conventional counts performed in the Neubauer chamber (Data presented in the supplementary information). A significant increase (p < 0.05) was observed in the production of reactive oxygen species 6 and 24 HPI in animals inoculated with bacteria in relation to those inoculated with saline (Fig. 5).

### Exudate

**Figure 6:**
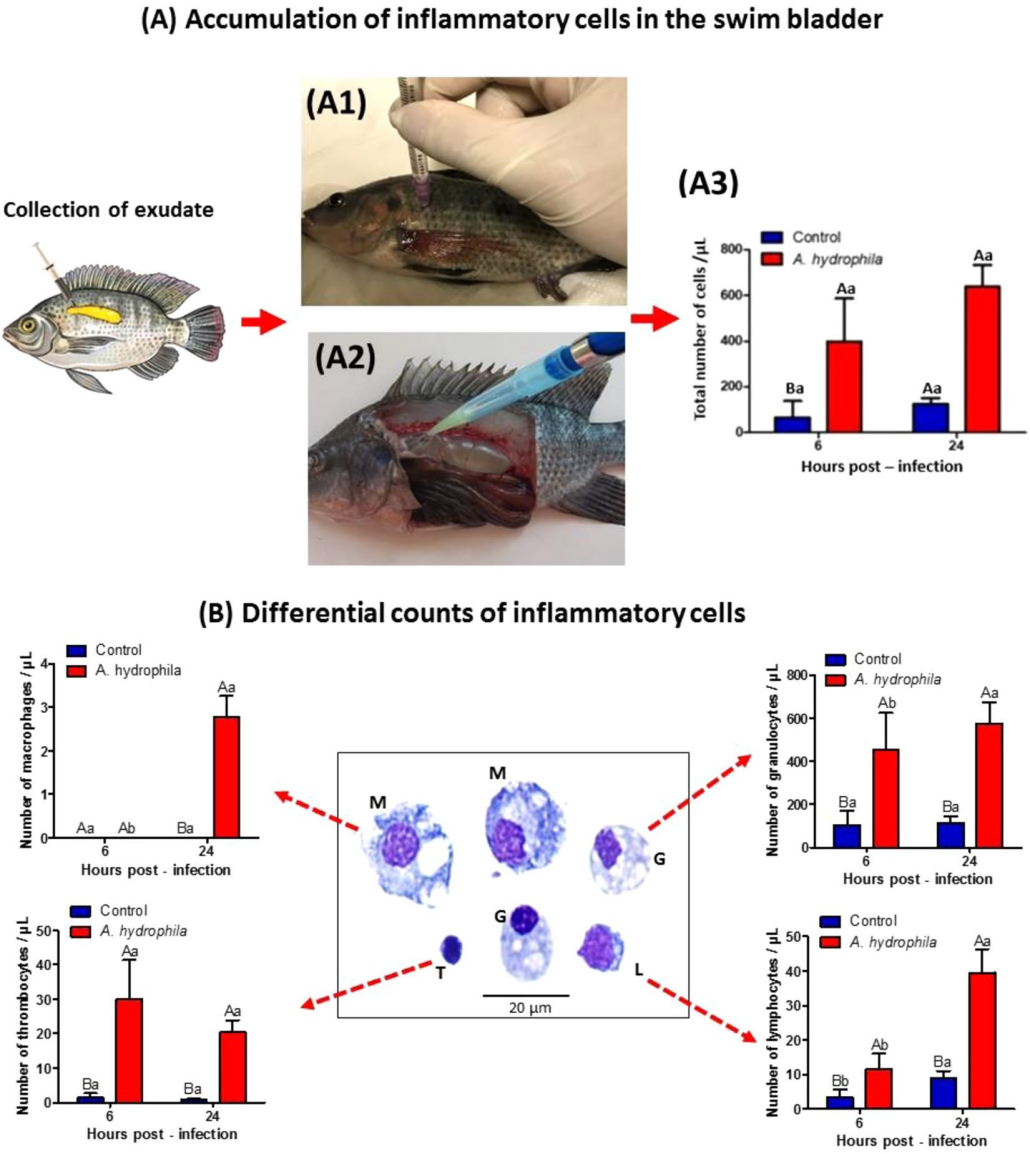
(**A**) Inflammatory cells presented in the exudate collected from swim-bladder. (A1) Anesthetized tilapia for bacterial inoculation in the swim bladder. (A2) At necropsy, collection of exudate from the swim bladder after lateral-left opening of the tilapia peritoneal cavities. (A3) Total cell counts observed in the exudate. (**B**) Differential counting of cells from the swim bladder exudate, and the morphology of cells in the inflammatory lesion. “G” (Granulocytes showed elliptical nuclei with small nonstained cytoplasmic granules), “T” (Thrombocytes presented a small nucleus:cytoplasm ratio and nuclei appearing mainly at the cell’s perimeter), “L” (Lymphocytes, small and spherical cells, with basophilic cytoplasm and apparently without granulations) and “M” (Macrophages, the largest cells observed in the exudates and exhibited cellular pleomorphism). Stained by May-Grünwald-Giemsa-Wright. Mean values (n = 10) and ANOVA observed for total cells and differential counting of cells present in the exudate during acute inflammatory response in tilapias 6 and 24 HPI. Means followed by the same letter do not differ by the Tukey test (P < 0,05). The variance analysis is represented by capital letters to compare the different treatments within each experimental period, lowercase letters to compare the evolution of each treatment in the different experimental periods. Different letters indicate significant difference (p < 0.05).

The accumulation of cells in the exudates showed a significant increase (p < 0.05) between 6 and 24 HPI in infected fish (Fig. 6A), with granulocytes being the most present cell types accumulated in the inflamed site (Fig. 6B). In the control animals inoculated with saline no significant variations were observed in the cellular accumulation present in the swim bladder (Fig. 6A).

The differential cell count revealed a significant increase in the number of lymphocytes, macrophages, granulocytes and thrombocytes in tilapia with infectious aerocystitis when compared to control animals inoculated with saline (Fig. 6B). The study of inflammatory reaction evolution showed a significant increase (p < 0.05) in the accumulation of granulocytes, thrombocytes, lymphocytes and macrophages in the exudates of fish inoculated with *A. hydrophila* between 6 to 24 HPI (Fig. 6B). Saline-inoculated control fish did not show significant variations in cell counts between the periods analyzed (Fig. 6B).

### Bacterial quantification assay

**Figure 7:**
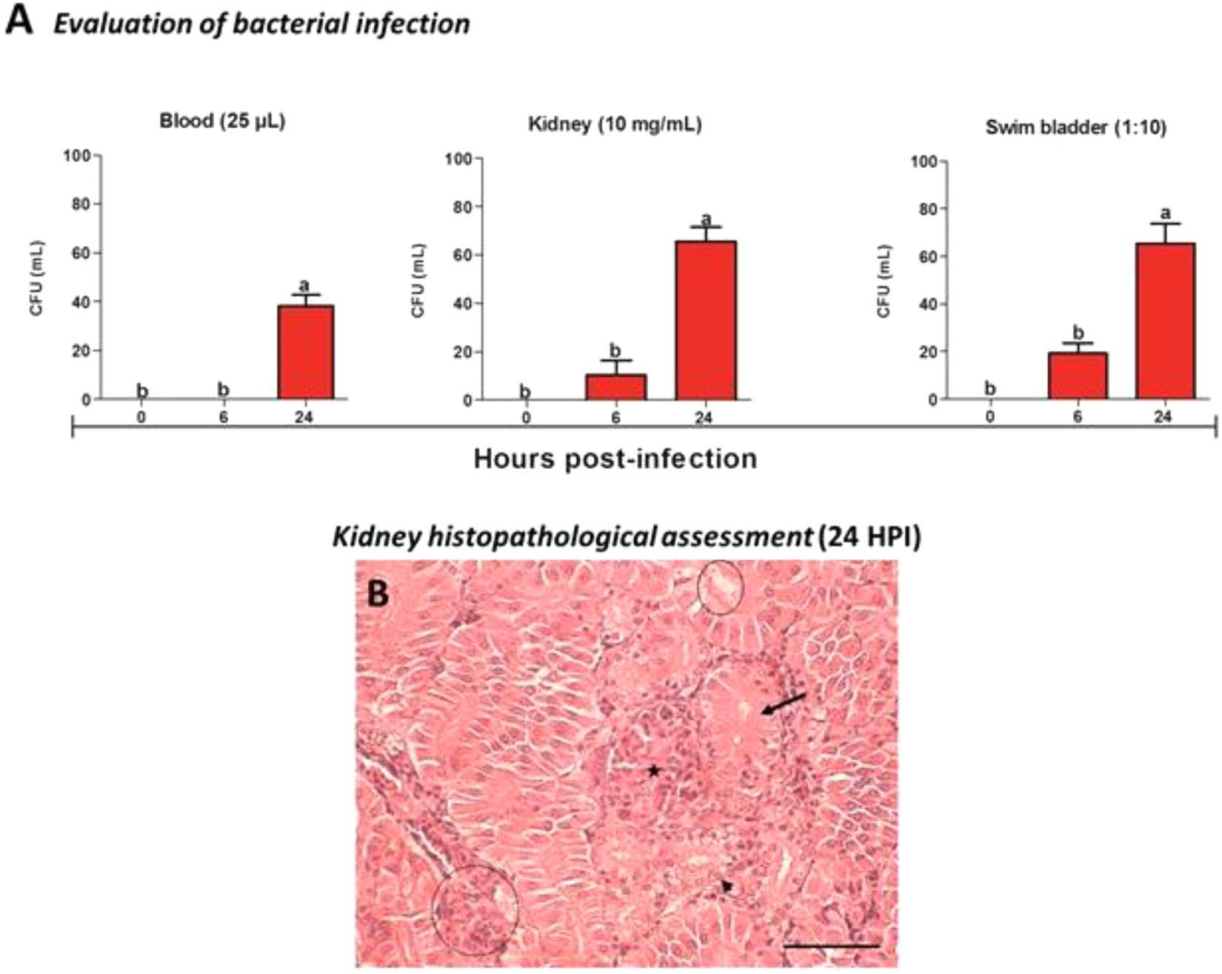
Bacterial infection (n = 10) and light micrographs of the caudal kidney of *O. niloticus* (n = 3). (**A**) Quantification of *A. hydrophila* in blood, caudal kidney and swim bladder. Mean values (n = 10) followed by the same letter do not differ by the Tukey test (P < 0,05). The variance analysis is represented by lower case letters. Different letters indicate significant difference (p < 0.05). (**B**) Kidney histopathological assessment (24 HPI), note the presence of epithelial detachment of tubular lumen (continuous circle), large obliteration of tubular lumen (arrow), increased glomerular tuft of Bowman capsule (star), hydropic degeneration in tubules (arrowheads), necrosis and inflammatory infiltrate (circle dotted). H&E, Bar 50 micrometers.

The quantitative results of the bacterium in the blood, caudal kidney and swim bladder for *A. hydrophila* are shown in Fig. 7A. The data showed that the model of swim bladder administration allowed bacterial dissemination through the blood and caudal kidney, including resulting in pathological changes in renal tissue which presented epithelial detachment of tubular lumen, large obliteration of tubular lumen, increased glomerular tuft of Bowman capsule, hydropic degeneration in tubules, necrosis and inflammatory infiltrate (Fig. 7B). The bacterial count of the caudal kidney every 10 mg/mL and the 10-fold diluted swim bladder were significantly increased (p < 0.05) at times of 6 and 24 HPI. However, no bacterial growth was observed in 25 μL sample of 6 HPI blood, and a significant increase (p < 0.05) 24 HPI. A negative control inoculated with saline solution was used in all experimental periods, and bacterial growth was negative.

## Discussion

The changes in protein fractions of tilapia during acute inflammatory reaction by infectious stimulus showed profiles similar to those observed in humans^8^, with increase of positive APPs such as ceruloplasmin, haptoglobin, alpha-2-macroglobulin and component C3 of the complement system, as well as decrease of negative APPs such as albumin and transferrin.

Like mammals, inflammatory cells such as macrophages and neutrophils release various cytokines such as IL-1, IL-6, IL-8 and TNF-α into the bloodstream in response to injury and tissue damage, which stimulate fish hepatocytes to produce the APPs and release them for circulation^2^. According to Vandevyver *et al*.^22^, acute phase responses are mainly regulated by glucocorticoids and IL-6. Blood levels of APPs change rapidly depending on the intensity and type of the stimulus^23^.

Among the proteins that were strongly influenced by the *A. hydrophila* infectious stimulus in tilapias, the multifunctional protein with high molecular weight α2-macroglobulin (95 to 115 kDa) showed a significant increase in its serum levels. In crustaceans, the α2-macroglobulin has a biological role in the blood coagulation system and phagocytosis^24^, while in mammals it inactivates during sepsis neutrophilic proteases, fibrinolysis-inhibiting proteases such as plasmin, and coagulation inhibitors such as thrombin, in addition to acting on metalloproteinases^25^.

α2M binds blood iron, zinc and copper ions stronger than albumin, and is known as transcupreins (serum copper transporters) by chelating copper ion in the human blood^26,27^. The acquisition of metal ions by microorganisms is essential for their survival in the infected host by the fact that these ions participate in biological processes as components of metalloproteins and serve as cofactors or structural elements for enzymes^28^. In many cases, the virulence of pathogenic bacteria are associated with the capture of iron, zinc, manganese and copper ions^28^. For these authors, part of the host defense strategies against infection consists of sequestering these metal ions.

*A. hydrophila* infection in tilapias resulted in a significant increase in the blood levels of ceruloplasmin and haptoglobin in the acute phase. Responsible for transporting more than 95% of the total copper in the bloodstream of healthy humans, ceruloplasmin is the main copper protein carrier in the blood^29^. In addition, plays a role in iron metabolism by exhibiting copper-dependent oxidase activity associated with oxidation of Fe^2+^ (ferrous iron) in Fe^3+^ (ferric iron), aiding in its transport in the blood associated to transferrin, which carries iron only in the ferric state^30^.

Haptoglobin in the blood binds to free hemoglobin^31^ and sequesters the iron present in this metalloprotein, preventing bacteria benefit of erythrocyte hemolysis in order to use the iron ions. In the case of tilapia with *A. hydrophila* infection, the increase in serum levels of α2M, haptoglobin and ceruloplasmin demonstrates the fish organism strategy to minimize the availability of these metal ions to bacterial metabolism, considering that this disease causes hemorrhagic septicemia and hemolytic anemia.

In this study, the dissemination of *A. hydrophila* in the blood and renal tissues, associated to the decrease in the number of circulating erythrocytes during the evolution of the infectious disease corroborate the positive APP findings. According to Li & Lu^32^, α2M was able to inhibit the extracellular protease of *A. hydrophila* (AhECPase) in carp, for these authors this APP may play a role in the resistance to infection by this pathogen. Bapat *et al*.^7^ observed a significant increase in α2M and haptoglobin expression in cases of *Mycobacterium tuberculosis* infection in humans. Enrichment of α2M with microparticles satisfactorily modulated the immune response and survival in human sepsis^33^.

Innate immune system activation is critical for host defense against invading microorganisms and for the subsequent generation of adaptive immune response, and proinflammatory mediators such as IL-6, which is considered key element in the pathogenesis of sepsis with regulation of APPs in the liver^34^. During the evolution of the acute phase response in mammals, the C3 component of the complement increases about 50%^35^, and this protein plays a central role in the complement system and contributes to innate immunity. Humans with C3 deficiency are susceptible to bacterial infections^36^. Tilapias infected with *A. hydrophila* showed a significant increase of C3 levels, demonstrating the same response profile observed in mammals.

Other studies have reported the involvement of APPs in the host immune response, α2M in humans is capable of binding to a range of cytokines including TNF-α and IL-1β, which play a key role in the inflammatory response^22^ and haptoglobin demonstrates anti-inflammatory activity by binding to CD11b/CD18 integrins, suggesting that this APP can regulate MAC-1 dependent cellular function *in vivo*^37^.

In the present study, there was a reduction in serum levels of albumin and transferrin in the acute phase response of tilapias infected with *A. hydrophila*, corroborating the findings of Güleç & Cengizler^23^, who observed a decrease in circulating levels of transferrin in tilapia during the first week after infection by *Streptococcus iniae*. In the inflammatory reaction, there is down-regulation of transferrin receptors, possibly through control mechanisms by regulatory proteins such as interferon gamma that results in decreased expression of this receptor in human mononuclear phagocytes^38^. Transferrin and its receptor are key and limiting factors in immune cell growth due to its iron requirement^39^, so that iron deficient rats reduced T cell proliferation and immunity was impaired^40^.

The liver is essential for protein homeostasis, being the primary site for the synthesis of many proteins, among them albumin considered to be the main hepatic plasma protein with a significant participation in intravascular oncotic pressure^41^. According to Ceciliani *et al*.^42^, there are no clear reasons to explain the down-regulation of some proteins during the acute phase response. One hypothesis to explain the significant decrease in albumin during inflammation would be the metabolic deviation to the synthesis of positive APPs, which would require the available amino acids in the proteins biosynthesis directly involved with the defense response. Hypoalbuminemia due to inflammatory processes are common findings in hospitalized humans^8^, which are aggravated in malnourished patients or with renal disorders^43^.

The homology between protein response profile of tilapia and mammals during the acute phase, for both positive and negative APPs, can be explained in part by the genetic proximity observed in the study of structural similarity between fish and human proteins. According to Howen *et al*.^44^, the teleost fish *Danio rerio* has its genome sequenced and comparison to the human reference genome shows that approximately 70% of human genes have at least one obvious zebrafish orthologue. This genetic proximity also reflects on the particularities and similarities of the fish defense responses when submitted to infectious aerocystitis.

The APP response of tilapias during the course of bacterial infection showed correlation with the kinetics of cellular accumulation in the swim bladder. The cellular component evaluated in the tilapia inflamed focus revealed greater cellular accumulation 24 HPI, corroborating the observations described by Claudiano *et al*.^11^ and Castro *et al*.^12^. The acute inflammatory reaction induced by *A. hydrophila* in tilapias resulted in a significant increase of granulocytes, thrombocytes, lymphocytes and macrophages accumulation when compared to the control animals inoculated with saline. However, granulocytes were the predominant cells in the inflamed focus during the acute phase similar to that observed in mammals.

In response to inflammatory stimuli, neutrophils migrate from the circulating blood to infected tissues, where they efficiently bind, engulf, and kill the bacteria through proteolytic enzymes, antimicrobial proteins, and reactive oxygen species (ROS)^45^. Likewise, tilapia infected with *A. hydrophila* showed a decrease in the number of circulating neutrophils associated to significant increase of granulocyte accumulation in the inflamed focus, demonstrating the kinetics of cellular recruitment from blood compartment to the infected tissue, associated to increase in the amount of C3 component from complement system and increment in the ROS production, suggesting increased respiratory burst activity of circulating leukocytes.

One important source to generate ROS are the NADPH oxidases present especially in phagocytes, such as polymorphonuclear neutrophils (PMNs)^46^. The teleost fish *Piaractus mesopotamicus* showed increment in ROS production with high neutrophil and monocyte counts, as well as increased phagocytic activity^47^. These fish presented higher survival rates during experimental infection with *A. hydrophila*.

Forn-Cuní *et al*.^48^ have demonstrated that the inflammatory response of teleostean fish can effectively reproduce the inflammatory process of mammals, showing that the signaling pathways and gene expression are well conserved throughout evolution. In this context, the search for alternative experimental models that demonstrate physiological responses similar to those observed in humans has become a very relevant subject in recent years, and countless researches have used fish as an experimental model to study the pathophysiology of inflammatory diseases with an interest in human medicine^49–56^.

Therefore, the modulation of positive and negative APPs observed in tilapias following inflammatory stimulus by the inoculation of *A. hydrophila*, and the cellular components present in exudates, as well as ROS production demonstrate the feasibility of this alternative experimental model for advances and studies to understand changes in pathophysiological mechanisms of acute inflammatory reaction due to bacterial infection.

## Material and Methods

### Experimental design

The experiment was carried out at São Paulo State University. A total of 40 Nile tilapia (*O. niloticus*) with average weight of 140 ± 10.2 g (SD) from the same spawn were allocated in 4 tanks, each with 250 L capacity (n = 10), with continuous water supply at a flow rate of 1 L/min and supplementary aeration, constituting the treatments: inoculated with *A. hydrophila* and inoculated with saline solution (to be sampled 6 and 24 hours after inoculation). The fish were acclimated for 15 days and fed with commercial feed (3% biomass, 28% GP and 4000 kcal of GE kg^−1^). Water quality parameters were examined daily at 7:30 am and 5:30 pm (pHmeter with condutivimeter YSI-63 and oximeter YSI-55), and the quality of the water remained within the adequate range for tropical fish comfort (dissolved oxygen = 5.35 ± 0.71 mg/L: temperature = 28.25 ± 1.92 °C; pH = 7.52 ± 0.83; and conductivity = 114.81 ± 11.54 μS/cm). This study was approved by the Ethics Committee for Use of Animals (CEUA) of São Paulo State University (UNESP), Jaboticabal (no. 11.860/16). The experiment was performed in accordance with relevant guidelines and regulations.

### Fish Anesthesia

The fish were anesthetized by immersion in 1:10,000 aqueous solution of benzocaine (sedation of the animals for administration of the bacterial inoculum) and 1: 500 (euthanasia moment). Initially, the benzocaine was diluted in 98° alcohol (0.1 g/mL) and the volume was filled to 1L^57^.

### Acute aerocystitis induced

The acute inflammation was induced by bacterial infection with *A. hydrophila* in the swim bladder isolated from naturally infected *O. niloticus* specimens, belonging to Pathology Laboratory of Aquatic Organisms, Aquaculture Center (UNESP). To confirm the species, DNA extraction and polymerase chain reaction (PCR) were performed to amplify the 16 S rDNA gene with subsequent sequencing. The sequences obtained were visualized and manipulated in the program Sequencer 5.0 (Gene Codes Corporation) at the Center of Biological Resources and Genomic Biology - Crebio (UNESP, Jaboticabal, SP). With the BLASTn tool, the fragment was compared to the sequences deposited in GenBank. The result of the sequencing confirmed the species *Aeromonas hydrophila*. On day zero, using 1.0 mL insulin syringes, fish were inoculated into the swim bladder with 0.5 mL of the *A. hydrophila* inoculum at the concentration of 3 × 10^9^ CFU/mL.

### Total Protein and albumin concentration

Fish blood samples were obtained by puncture from the caudal vessel and placed in 2.0 mL microtubes, without anticoagulant, centrifuged at 3000 g for 10 minutes at 4 °C, and separated the serum for total protein and albumin determination using in a semi-automatic biochemical analyzer (Model LabQuest® – Bioplus Company)^41^.

### SDS-PAGE protein electrophoresis

After 6 and 24 h, protein content was assessed by means of electrophoretic fractioning in a vertical system using the SDS-PAGE method, with the gel prepared as described by Laemmli^58^. Molecular weight and protein fraction levels were determined using readings from a computerized densitometer (Shimadzu CS-9301, Tokyo, Japan). To identify the protein content, different markers of molecular weights, ranging from 20 to 200 kDa.

### In-gel protein digestion and mass spectrometric identification

Protein bands were excised from the SDS-polyacrylamide and in-gel trypsin digestion was performed according to Shevchenko *et al*.^59^. Mass spectrometric analysis by LC–MS/MS. Peptide samples (4.5 μL) were automatically injected into a trap column packed with C18 (180 µm id × 20 mm) (Waters, Milford, MA) for desalting with 100% solvent A (0.1% formic acid) at 15 µL/min for 3 min. Peptides were then eluted onto an analytical C18 column (100 µm id × 100 mm) (Waters, Milford, MA) using a 20 min gradient at a flow rate of 600 nL/min where solvent A was 0.1% formic acid and solvent B was 0.1% formic acid in acetonitrile. The gradient was 0–3% of solvent B in 1 min, 3–60% B in 14 min, 60–80%B in 2.5 min, 80%B for 1 min, then back to 3%B in 1.5 min. An ESI Q-TOF Ultima mass spectrometer (Waters, Milford, MA) was used to acquire spectra. Spray voltage and temperature were set at 3.4 kV and 100 °C, respectively, and the instrument was operated in data dependent mode, in which one full MS scan was acquired in the m/z range of 200–2000 followed by MS/MS acquisition using collision induced dissociation of the three most intense ions from the MS scan. Dynamic peak exclusion was applied to avoid the same m/z to be selected for the next 45 s.

Raw data files of fragment spectra were processed by ProteinLynx 2.2 (Waters, Milford, MA) and converted to *pkl format. The resulting fragment spectra were searched using Mascot (version 2.4.1) search engine (Matrix Science, UK) against the UniProt and NCBInr database restricted to Actinopterigii Class with a parent tolerance of 1.5 Da and fragment tolerance of 1.0 Da. Iodoacetamide derivative of cysteine and oxidation of methionine were specified in Mascot as fixed and variable modifications, respectively. Mascot identifications required ion scores greater than the associated identity scores and 20, 30, 40 and 40 for singly, doubly, triply, and quadruply charged peptides, respectively.

### Analysis of protein structural similarity

For the protein comparative analysis among the species, all FASTA files related to tilapia proteins and other species were acquired on the UNIPROT search platform (http://www.uniprot.org) and the percentage of similarity between the species and their homologous proteins was processed on the EMBOSS Water platform (https://www.ebi.ac.uk). For 3D structural comparison, FASTA files were transcribed in PDB file (protein file in 3D structural form) using the tool Raptor X 9 (http://raptorx.uchicago.edu) and the structural similarities were compared on the iPDA platform (http://www.dsimb.inserm.fr).

### Evaluation of blood cells by flow cytometry

The total and differential counts of the blood cells were obtained through the technique of flow cytometry (FACScan - Becton Dickinson). Blood cells with membrane permeability compromised were detected by propidium iodide fluorescence. Briefly, aliquots of blood cells (25 μL) were incubated for 15 minutes with 3 μL of propidium iodide (50 µg/ml) (PI - Sigma) in the dark. The samples were suspended in 1 mL of PBS and immediately analyzed by flow cytometry as described above. The propidium iodide fluorescence was examined in 100,000 cells per animal. All FACS parameters (FSC and SSC) and region settings were the same throughout all experiments. The data were analyzed using FlowJo software®^60^.

### Cell sorting and light microscopy

Blood samples were previously diluted (20x) in phosphate-buffered saline containing 5% bovine serum albumin (BSA). Each sample was filtered in a cell strainer (40 µm - BD) and their populations were separated in different vials using a flow cytometer (BD-FACSAria III – Cell Sorting)^60,61^. A minimal of 100,000 cells was carried out for each sample^62^. The vials containing each population previously separated were analyzed again in FACSAria III in order to confirm the location of each one. Subsequently, smears were produced for acquisition of images under light microscopy. The smears were prepared on air-dried slides and stained with May-Grünwald solution for 6 min. The images of smear cells were acquired with an Olympus BX41 light microscope with a Q-color digital camera attached, using 100x objective^63^.

### Image stream

Images of the vials sample sorted by BD-FACSAria were performed in Image Stream®X Mk II – AMNIS cytometer. The acquisitions were carried out in bright field mode by using 40 x objectives. This equipment can acquire images of 2,000 cells per second using a field of view of 60 × 128 µm and obtains images with a pixel size of 0.5 µm.

### Blood cells quantification during infection

Total blood cell and differential cell counts were performed with a BD FACScan flow cytometer (Becton Dickinson) and data analysis were performed using the FlowJo Version 9.2 software®. The blood samples were resuspended in 1 mL of PBS and immediately analyzed by the flow cytometer. A total of 30,000 cells per animal were observed and all FACS parameters (FSC and SSC) and region settings were kept identical throughout all experiments. The data were analyzed using the FlowJo software®.

### Exudates cell component evaluation

After euthanasia 6 and 24 h, the exudate was collected by washing the swim bladder with 1.0 mL of PBS, containing 0.01 mL of 5% EDTA. The washing liquid was fully recovered and placed in conical test tubes, which were kept on ice and centrifuged at 1000 g in a refrigerated centrifuge at 4 °C for 5 min. The supernatant was removed and the sediment was suspended with the addition of known volume of the same phosphate buffer. An aliquot was transferred to a Neubauer chamber to count the total number of cells under an optical microscope, and another aliquot was used to prepare panchromatically stained smears using May–Grünwald–Giemsa–Wright^10^, and 200 cells were counted to establish the percentage of macrophages, granulocytes, thrombocytes and lymphocytes.

### Reactive Oxygen Species (NBT assay)

Blood samples were destined for the nitro blue tetrazolium (NBT) assay. 0.5 mL of blood was transferred to 2 mL plastic tubes containing 15 μL of heparin (5.000 IU mL−1). The respiratory burst of leukocytes was measured according to Farias *et al*.^47^. Briefly, 100 μL of heparinized blood was mixed with 100 μL of NBT (0.2% w/v PBS – Sigma, St. Louis, MO, USA). The solution was homogenized and incubated in a dark room for 30 min at 25 °C. After incubation, 50 μL of the solution was added in 5 mL tubes containing 1 mL of n,n-dimethylformamide (DMF, Sigma, St. Louis, MO, USA) and centrifuged at 3000 × g for 5 min. The optical density of the supernatant was measured using a spectrophotometer with optical density of 540 nm.

### Bacterial quantification during infection

The bacterial quantification assay was performed according to Li *et al*.^64^. Briefly, quantification of bacterial numbers was performed by the spread plate technique in TSA medium with ampicillin (10 mg/L). Exudate from the swim bladder, blood and caudal kidney were collected aseptically from fish 6 and 24 HPI. The caudal kidney was homogenized in 10 mg/mL sterile PBS (pH 7.2). The swim bladder was diluted 10-fold (1:10) in sterile PBS (pH 7.2) and plated. Whole blood was plated in the volume of 25 μL. After incubation for 18 h at 28 °C, the bacterial colonies were counted separately in colonies counter. Each sample was seeded in triplicate and the results expressed in colony forming units (CFU). Isolates from the bacterial recovery study were identified using the biochemical test for Gram negative and oxidase positive bacteria (Bactray III, Laborclin®, Pinhais Brazil). Fragments of kidney were collected and fixed with 10% formaldehyde in phosphate buffer. Subsequently, they were embedded in paraffin and then made 5-μm sections to be stained with hematoxylin and eosin (H & E) for the diagnosis of histopathological alterations^65^.

### Statistical analysis

The experiment was entirely randomized. Normality of the data was confirmed by the Shapiro-Wilk test. All data was statistically analyzed using a factorial scheme “Split-plot design” [two treatment (infected and control) X two periods (6 and 24 HPI)], according to Littell *et al*.^66^. The analysis of variance for comparing the different experimental groups was carried out by applying a General Linear Model (GLM) Procedure (SAS, 2012)^67^. Significant differences (P < 0.05) were estimated on the basis of Tukey test^68^.

## Supplementary information

Supplementary information
