# Supplementary material for: Acute-phase proteins during inflammatory reaction by bacterial infection: Fish-model
Source: Sci Rep. 2019 Mar 18;9:4776. doi: 10.1038/s41598-019-41312-z (PMC6423045; doi:10.1038/s41598-019-41312-z)
Supplement: Supplementary file 1 — Supplementary information [file 41598_2019_41312_MOESM1_ESM.pdf]

Supplementary Information

Acute-phase proteins during inflammatory reaction by bacterial infection: Fish-model

Ives Charlie-Silva[1], Andre Klein[2], Juliana M. M. Gomes[2]; Ed J. R. Prado[1]; Alessandra C. Moraes[1]; Silas F. Eto[1]; Dayanne C. Fernandes[1]; José J. Fagliari[1]; José D. Corrêa Junior[2]; Carla Lima[3]; Mônica Lopes-Ferreira[3]; Katia Conceição[4]; Wilson G. Manrique[5] & Marco A. A. Belo[1,6 *]

[1] Department of Preventive Veterinary Medicine, São Paulo State University (UNESP), Jaboticabal/SP, Brazil.

[2] Department of Pharmacology and Morphology, ICB-UFMG, Belo Horizonte/MG. Brazil.

[3] Laboratory of Immuno Regulation at Unit Butantan Institute, São Paulo/ SP – Brazil.

[4] Laboratório de Bioquímica de Peptídeos, UNIFESP, São José dos Campos/SP, Brazil.

[5] Veterinary College, Federal University of Rondonia, Rolim de Moura,RO, Brazil.

[6]Laboratory of Animal Pharmacology and Toxicology, Brasil University, Descalvado/SP, Brazil.

*** Corresponding author:**

Marco Antonio de Andrade Belo (PhD)

Laboratory of Animal Pharmacology and Toxicology, Brasil University. Av. Hilário da Silva Passos, 950. Jardim Universitário, Descalvado/SP, 13690-000, Brazil. Phone: +5501935938575. Email: maabelo@hotmail.com

Attachment 1

(SDS-PAGE polyacrylamide gel)

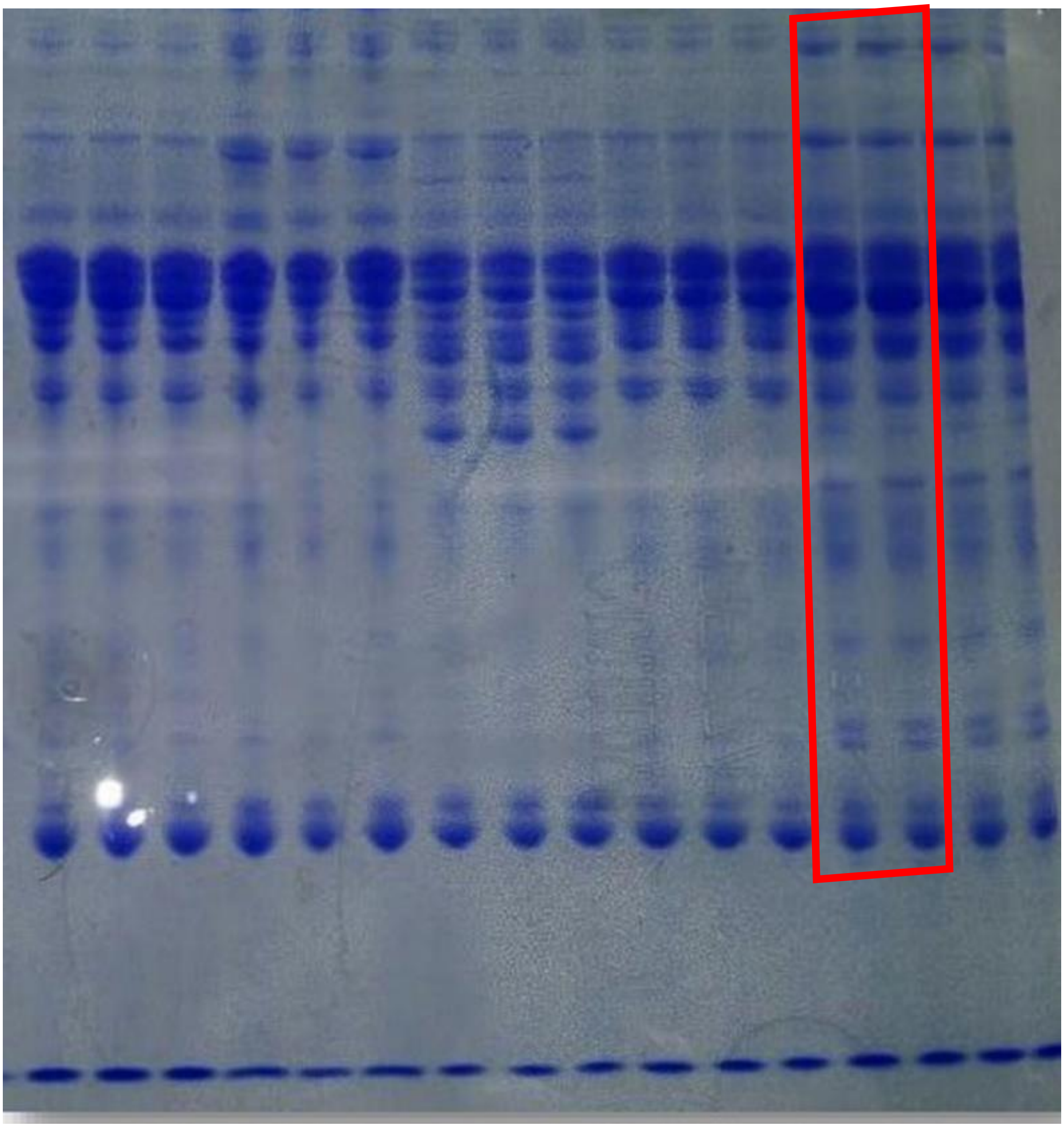

Figure I. SDS-PAGE polyacrylamide gel during acute inflammatory response in tilapias 6 and 24 HPI. Highlighted in red the area presented in the Figure 1.

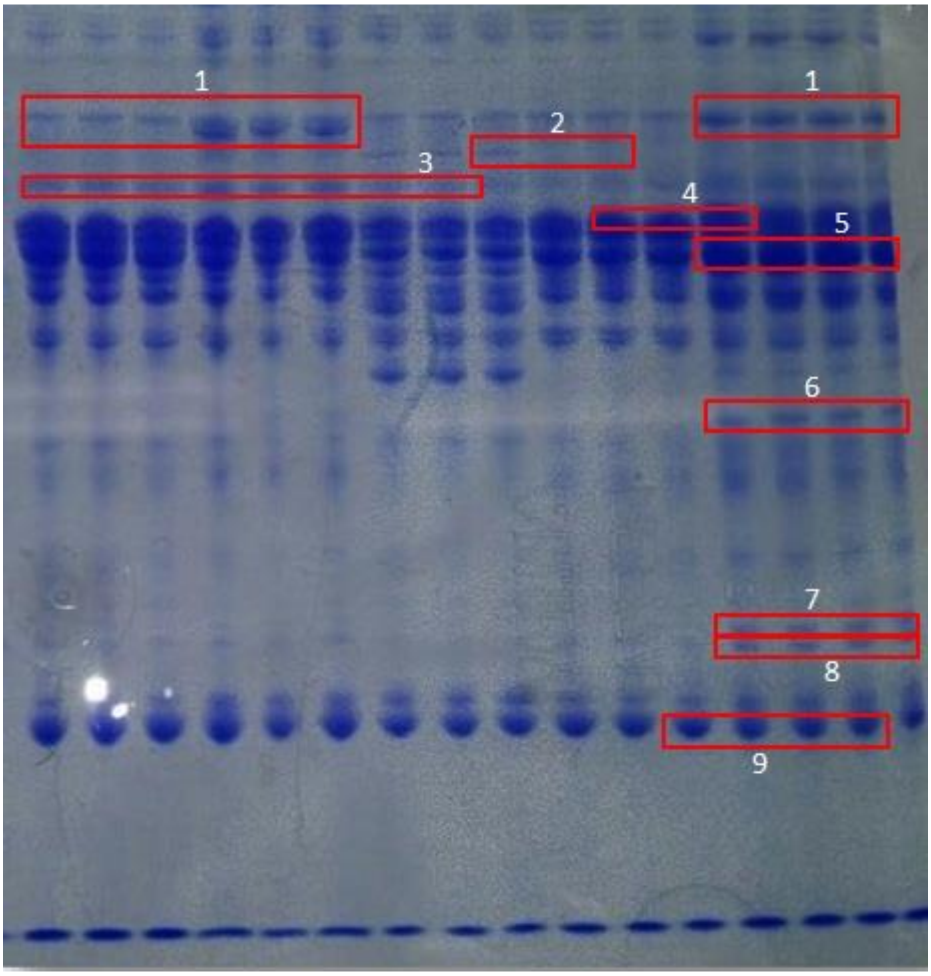

Figure II. SDS-PAGE polyacrylamide gel demonstrating the cut areas (highlighted in red) submitted to in gel trypsin digestion coupled to mass spectrometric analysis resulting in the identification of proteins present in the bands (Figure 1). (1) ceruloplasmin; (2) complement C3; (3) α_2 macroglobulin; (4) albumin; (5) transferrin; (6) haptoglobin; (7) apolipoprotein Eb and complement C3 isoform X1; (8) complement factor 3 and apolipoprotein Eb; (9) apolipoprotein A1.

Attachment 2

(Protein comparative analysis)

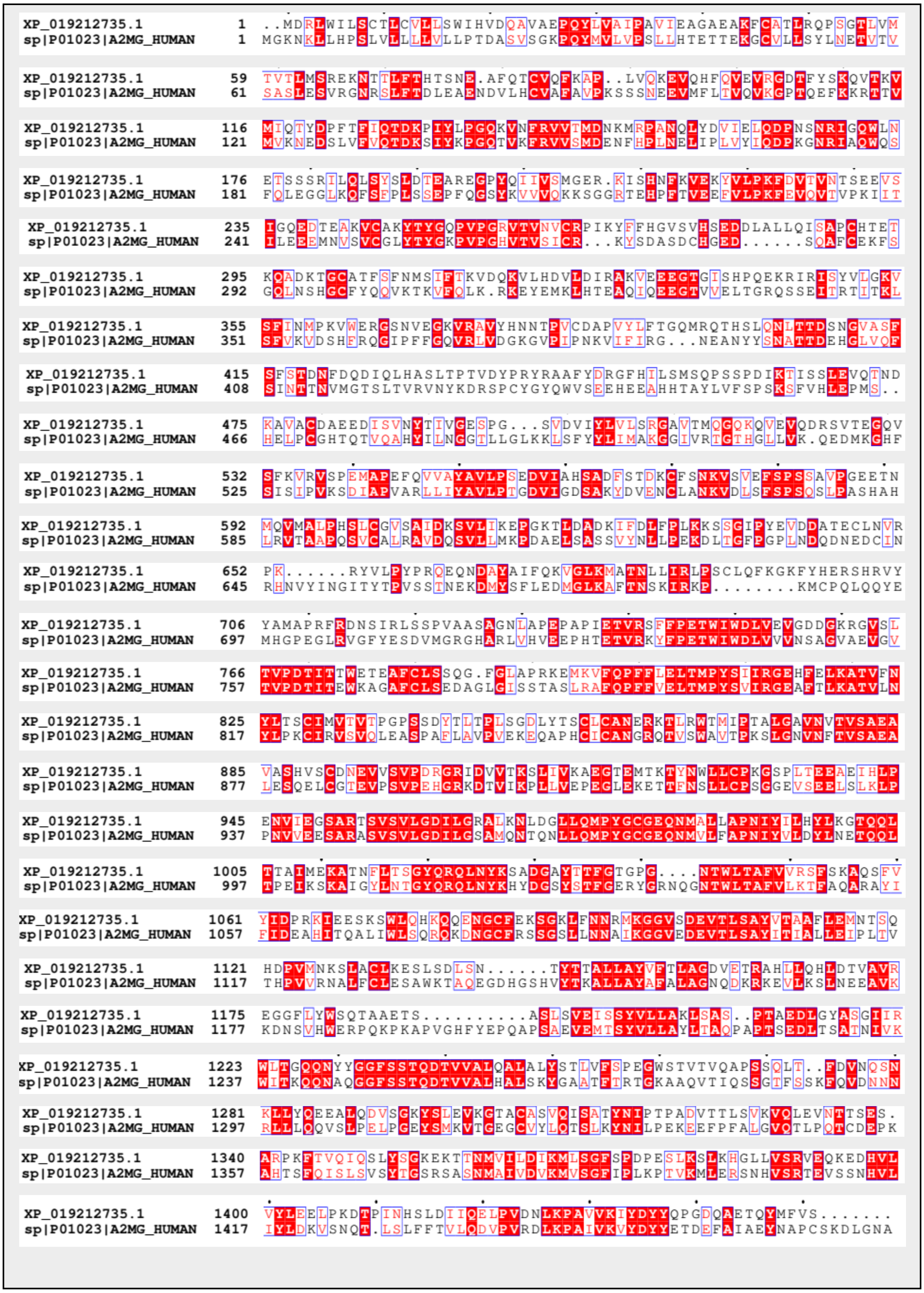

Figure III. Structural comparison of the alpha-2-macroglobulin between *Oreochromis niloticus* and *Homo sapiens*. The FASTA files related to tilapia and human proteins were acquired on the UNIPROT search platform (http://www.uniprot.org).

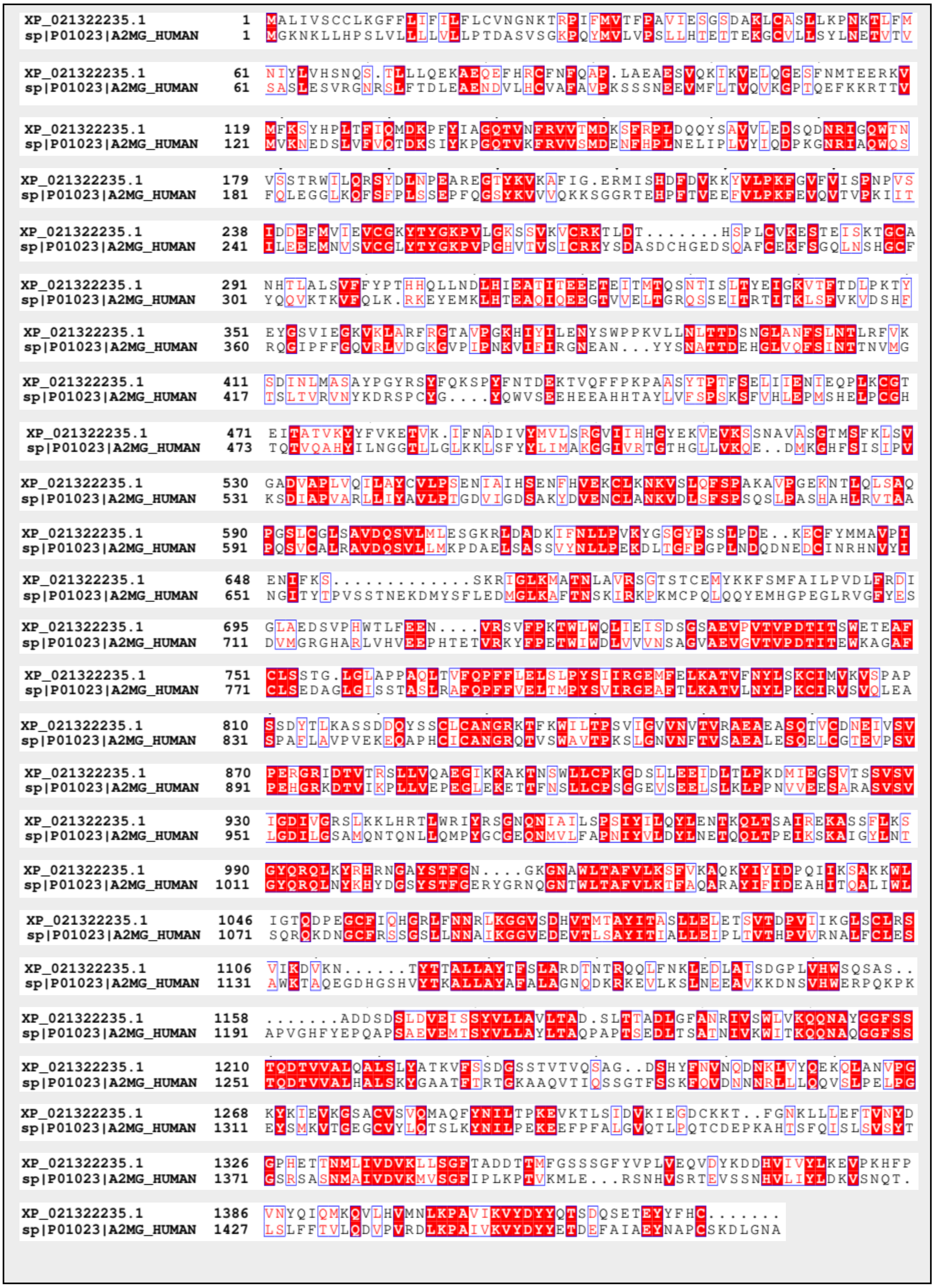

Figure IV. Structural comparison of the alpha-2-macroglobulin between *Danio rerio* and *Homo sapiens.* The FASTA files related to zebrafish and human proteins were acquired on the UNIPROT search platform (http://www.uniprot.org).

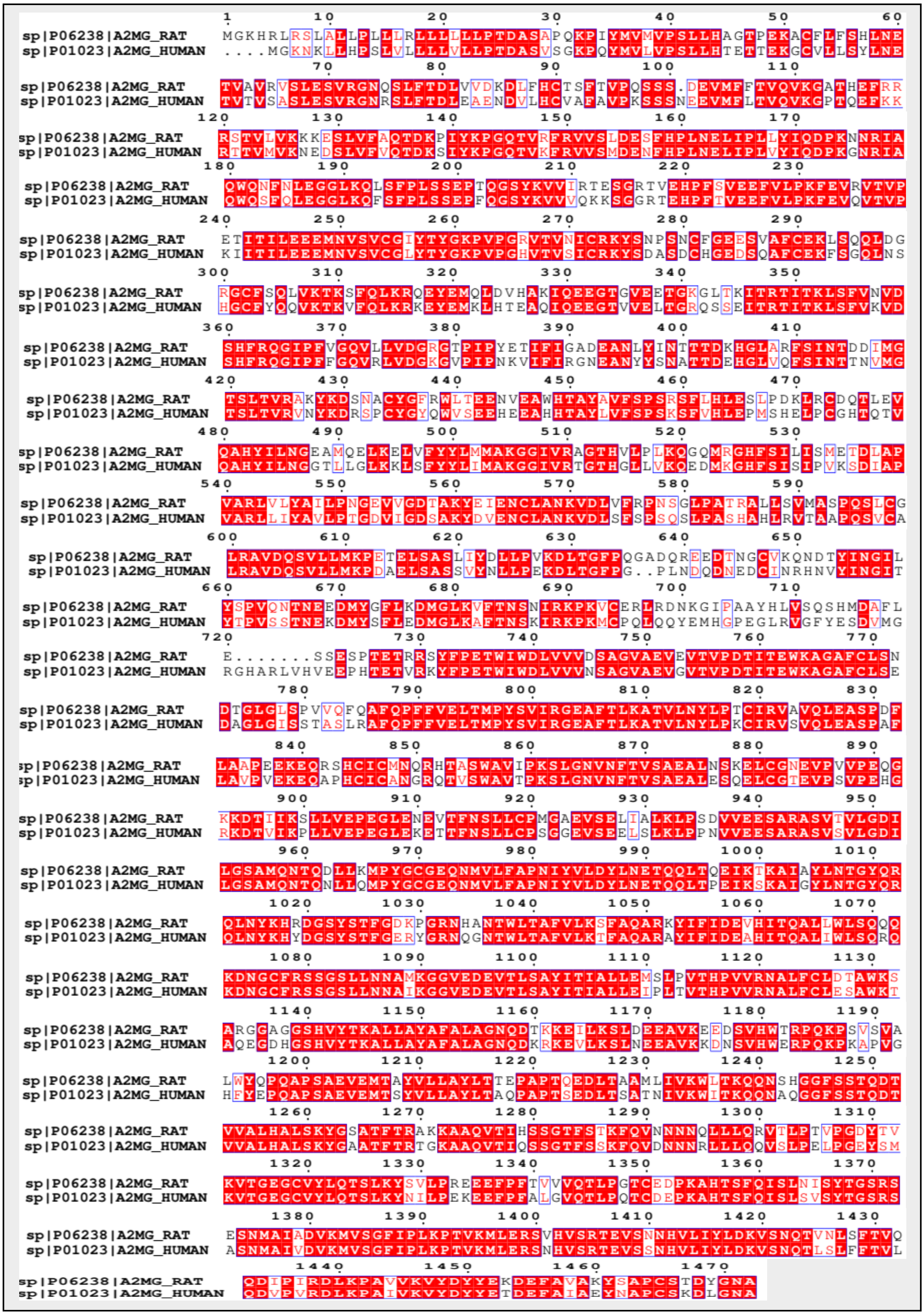

Figure V. Structural comparison of the alpha-2-macroglobulin between *Rattus norvegicus* and *Homo sapiens*. The FASTA files related to rodent and human proteins were acquired on the UNIPROT search platform (http://www.uniprot.org).

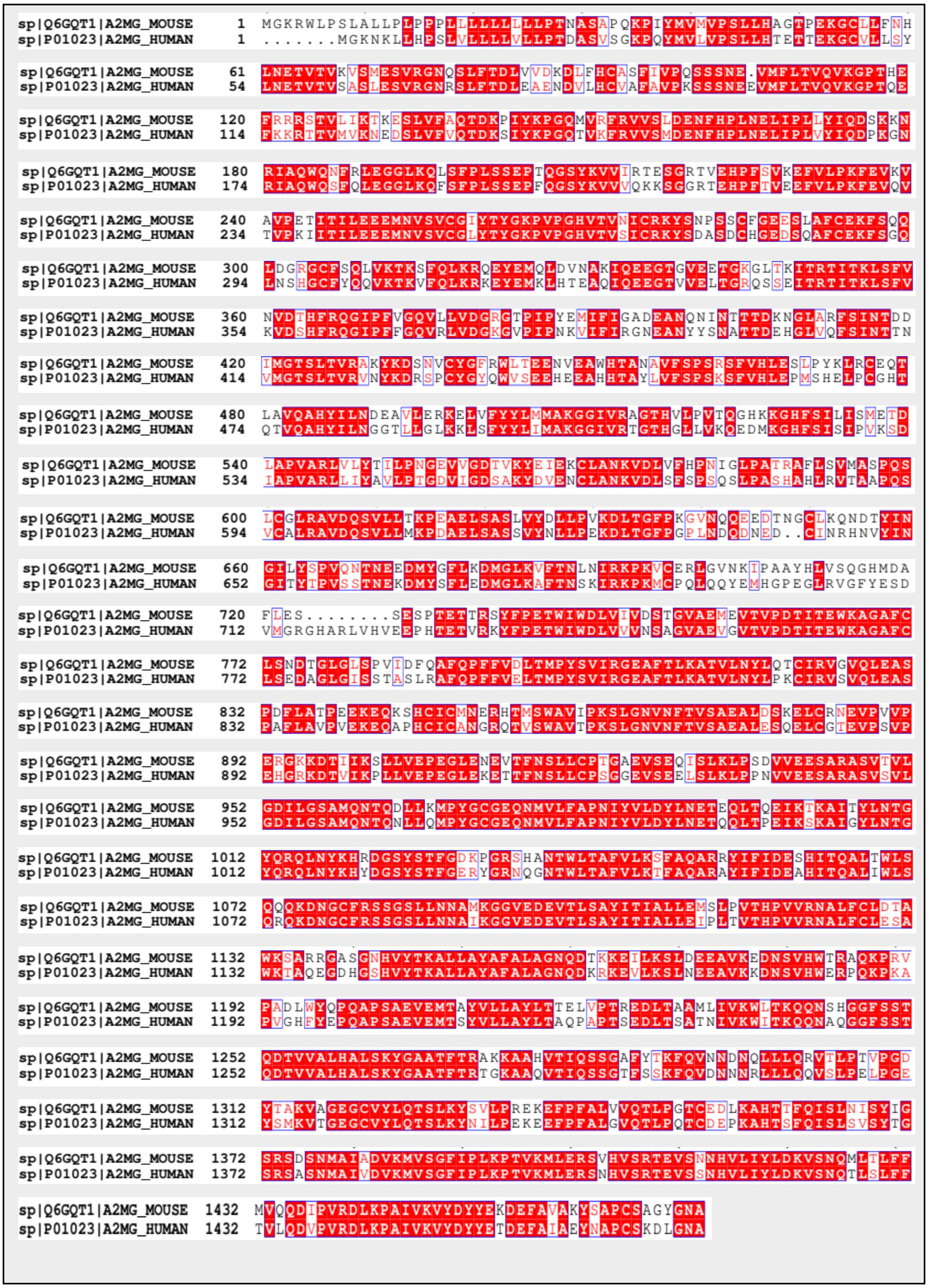

Figure VI. Structural comparison of the alpha-2-macroglobulin between *Mus musculus* and *Homo sapiens*. The FASTA files related to rodent and human proteins were acquired on the UNIPROT search platform (http://www.uniprot.org).

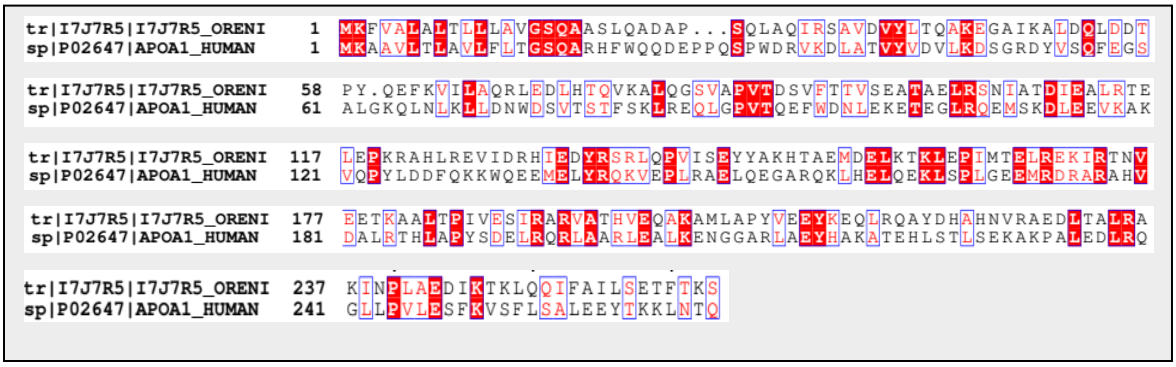

Figure VII. Structural comparison of the apolipoprotein A1 between *Oreochromis niloticus* and *Homo sapiens*. The FASTA files related to tilapia and human proteins were acquired on the UNIPROT search platform (http://www.uniprot.org).

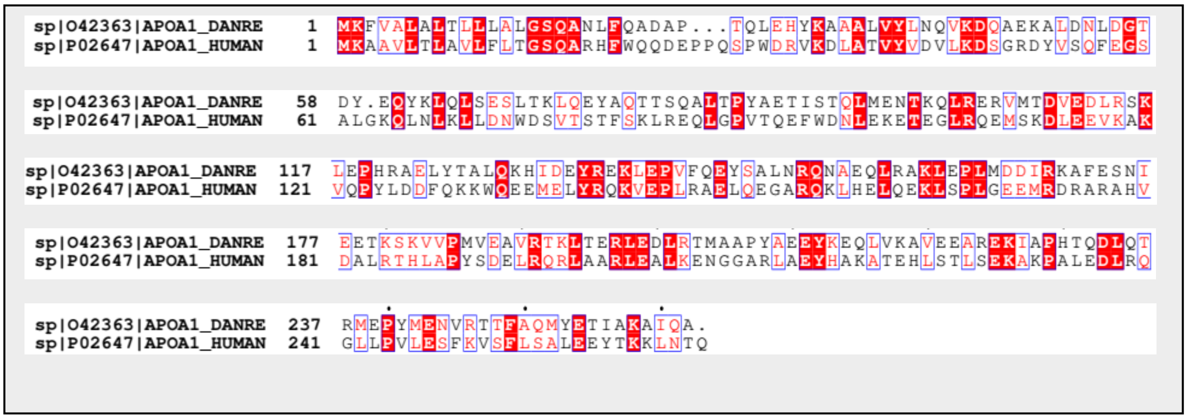

Figure VIII. Structural comparison of the apolipoprotein A1 between *Danio rerio* and *Homo sapiens*. The FASTA files related to zebrafish and human proteins were acquired on the UNIPROT search platform (http://www.uniprot.org).

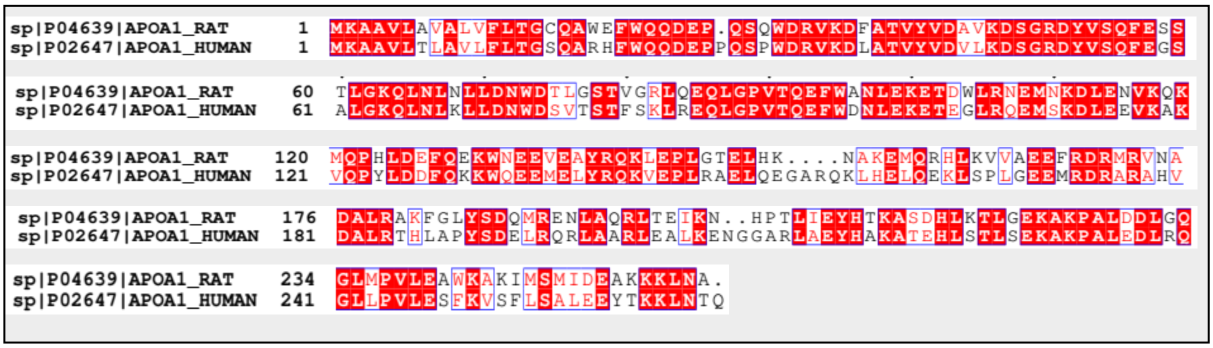

Figure IX. Structural comparison of the apolipoprotein A1 between *Rattus norvegicus* and *Homo sapiens*. The FASTA files related to rodent and human proteins were acquired on the UNIPROT search platform (http://www.uniprot.org).

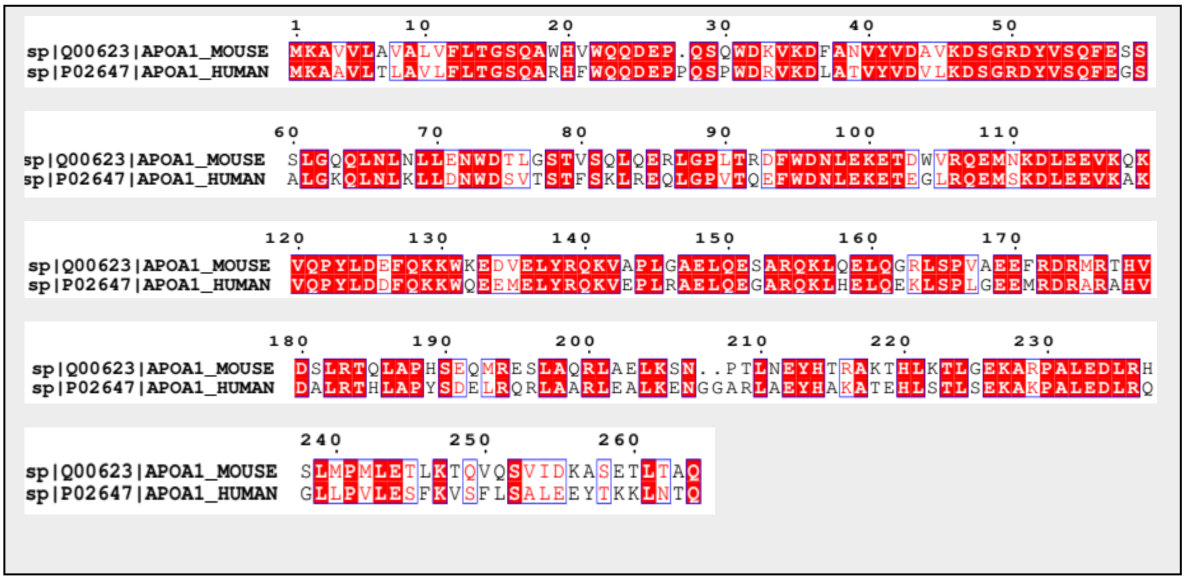

Figure X. Structural comparison of the apolipoprotein A1 between *Mus musculus* and *Homo sapiens*. The FASTA files related to rodent and human proteins were acquired on the UNIPROT search platform (http://www.uniprot.org).

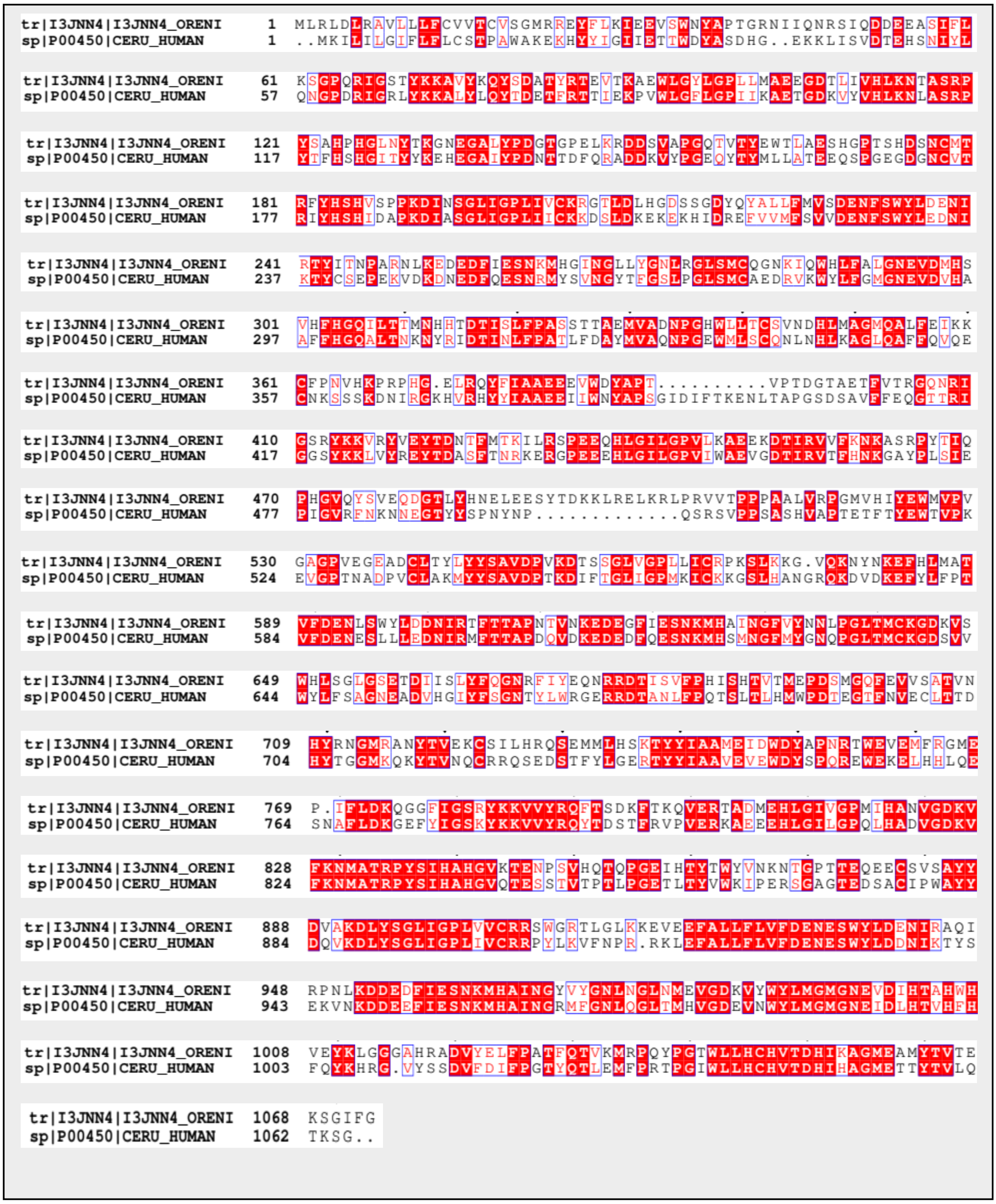

Figure XI. Structural comparison of the ceruloplasmin between *Oreochromis niloticus* and *Homo sapiens*. The FASTA files related to tilapia and human proteins were acquired on the UNIPROT search platform (http://www.uniprot.org).

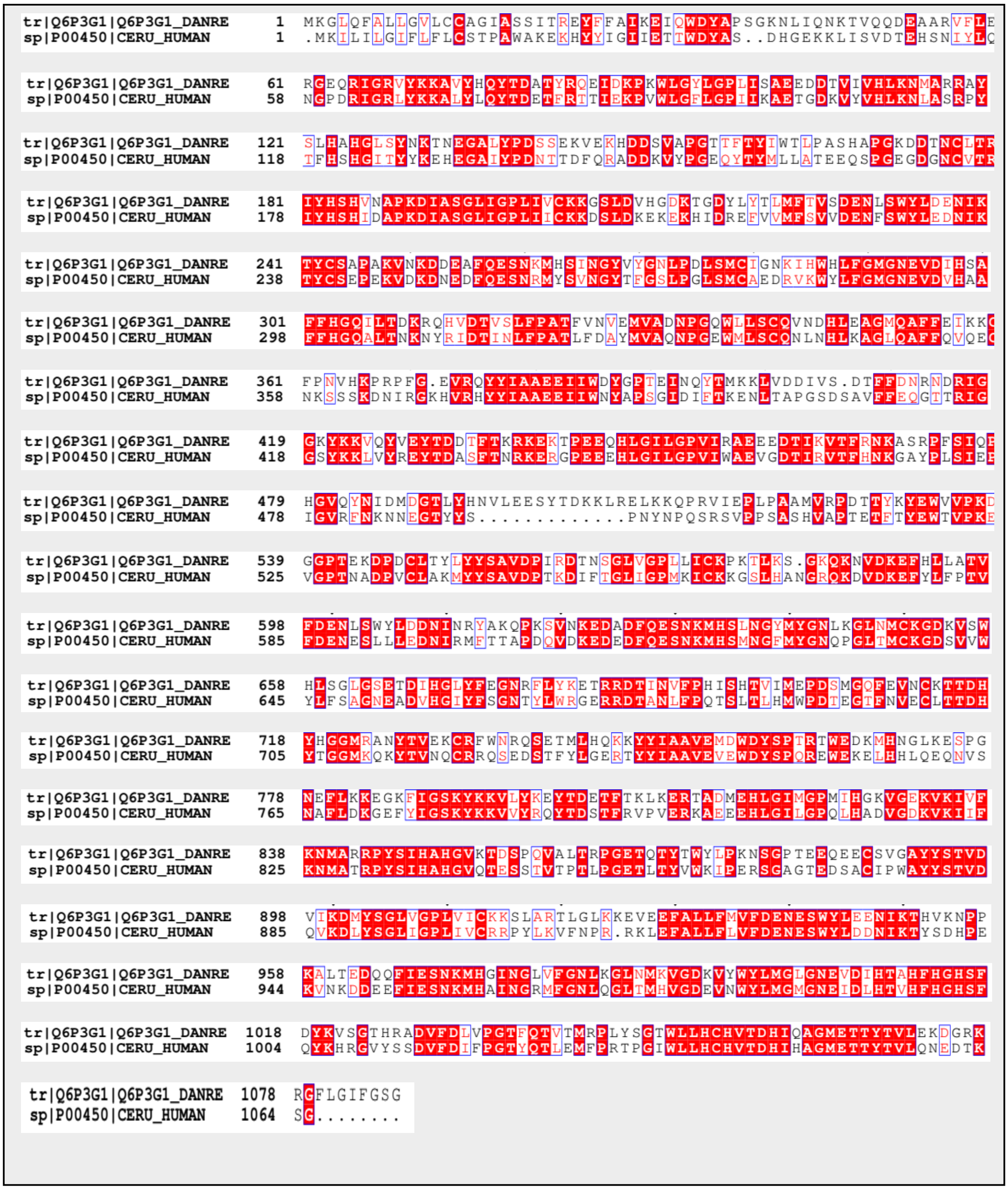

Figure XII. Structural comparison of the ceruloplasmin between *Danio rerio* and *Homo sapiens*. The FASTA files related to zebrafish and human proteins were acquired on the UNIPROT search platform (http://www.uniprot.org).

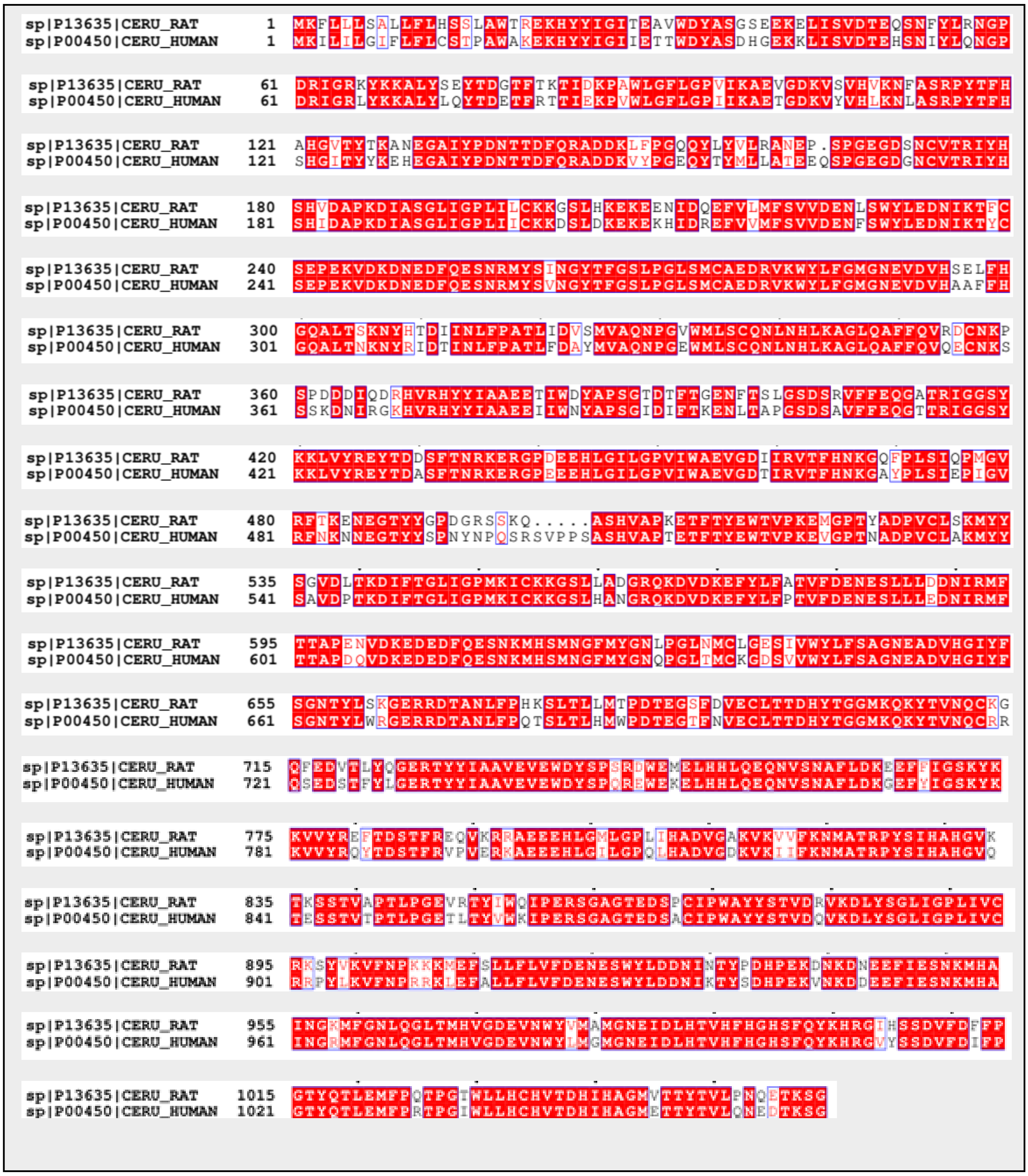

Figure XIII. Structural comparison of the ceruloplasmin between *Rattus norvegicus* and *Homo sapiens*. The FASTA files related to rodent and human proteins were acquired on the UNIPROT search platform (http://www.uniprot.org).

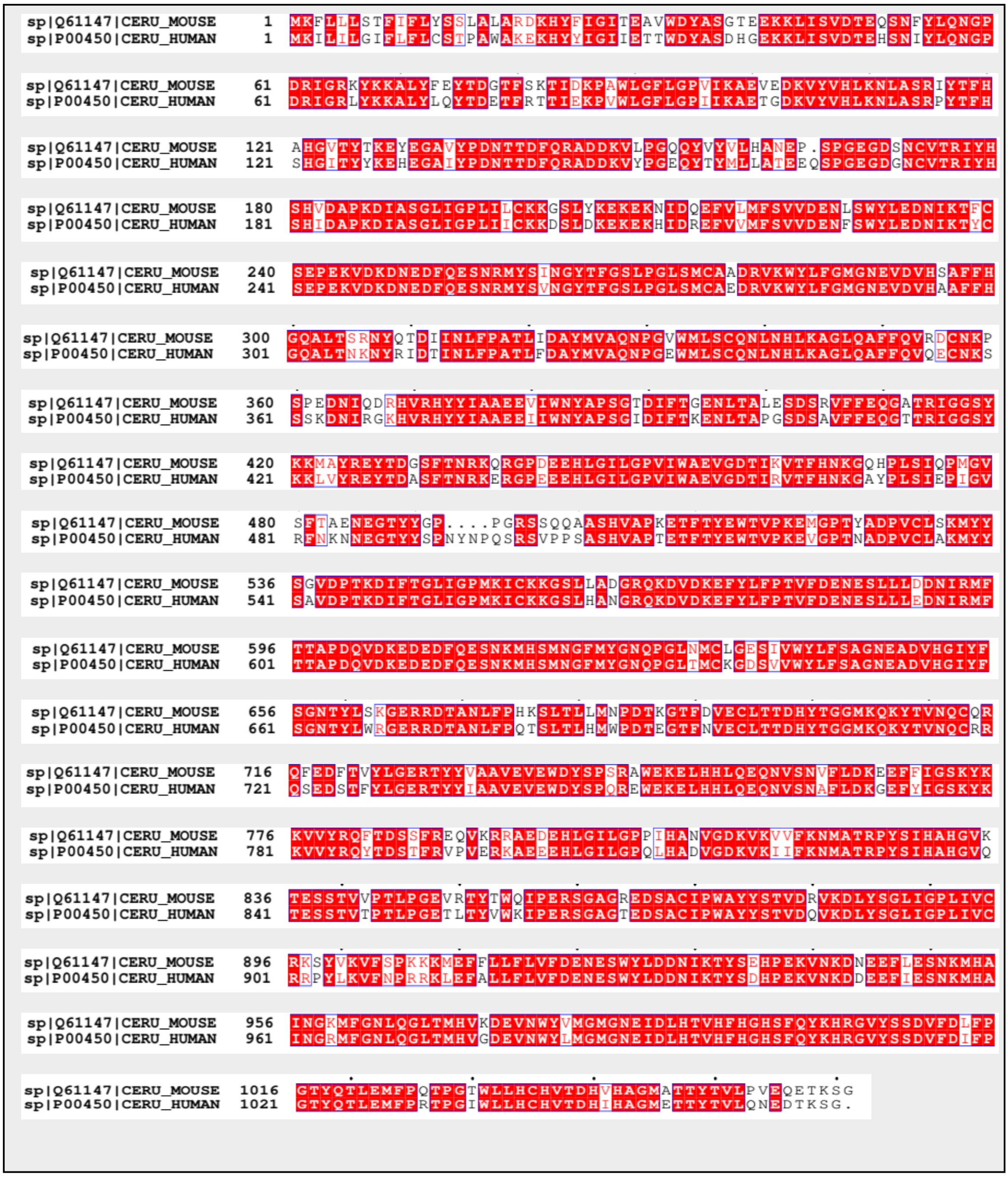

Figure XIV. Structural comparison of the ceruloplasmin between *Mus musculus* and *Homo sapiens*. The FASTA files related to rodent and human proteins were acquired on the UNIPROT search platform (http://www.uniprot.org).

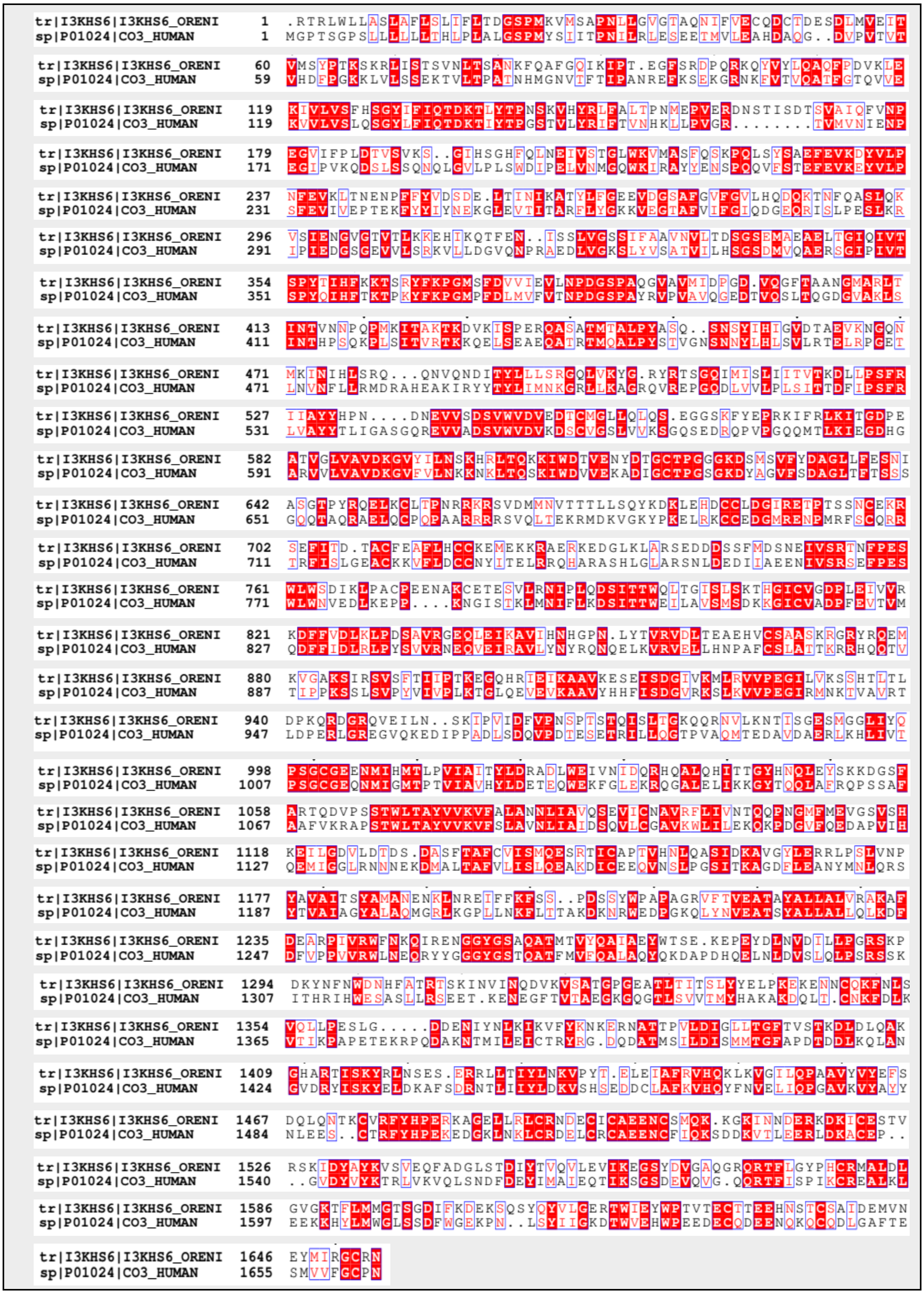

Figure XV. Structural comparison of the complement C3 between *Oreochromis niloticus* and *Homo sapiens*. The FASTA files related to tilapia and human proteins were acquired on the UNIPROT search platform (http://www.uniprot.org).

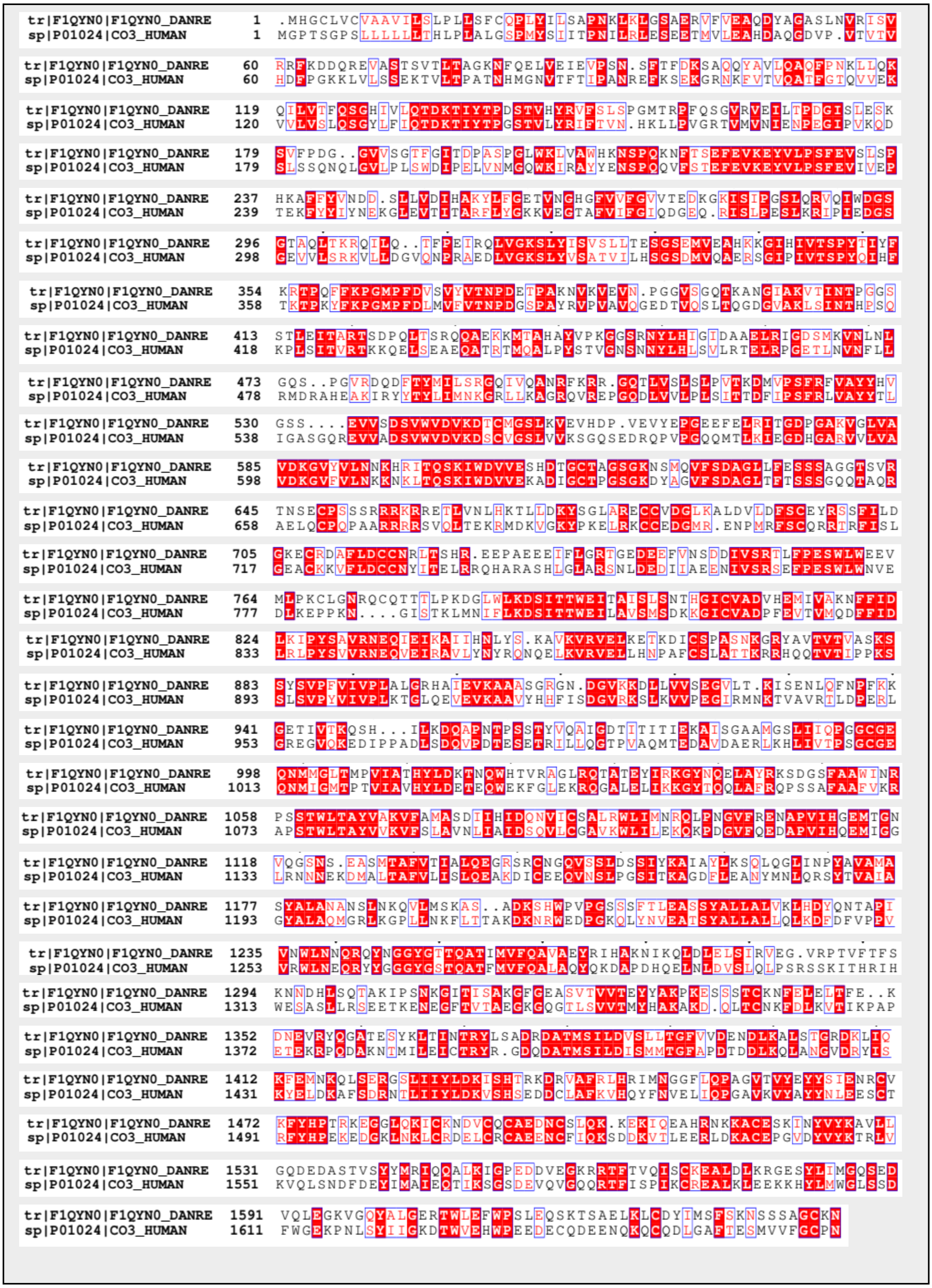

Figure XVI. Structural comparison of the complement C3 between *Danio rerio* and *Homo sapiens.* The FASTA files related to zebrafish and human proteins were acquired on the UNIPROT search platform (http://www.uniprot.org).

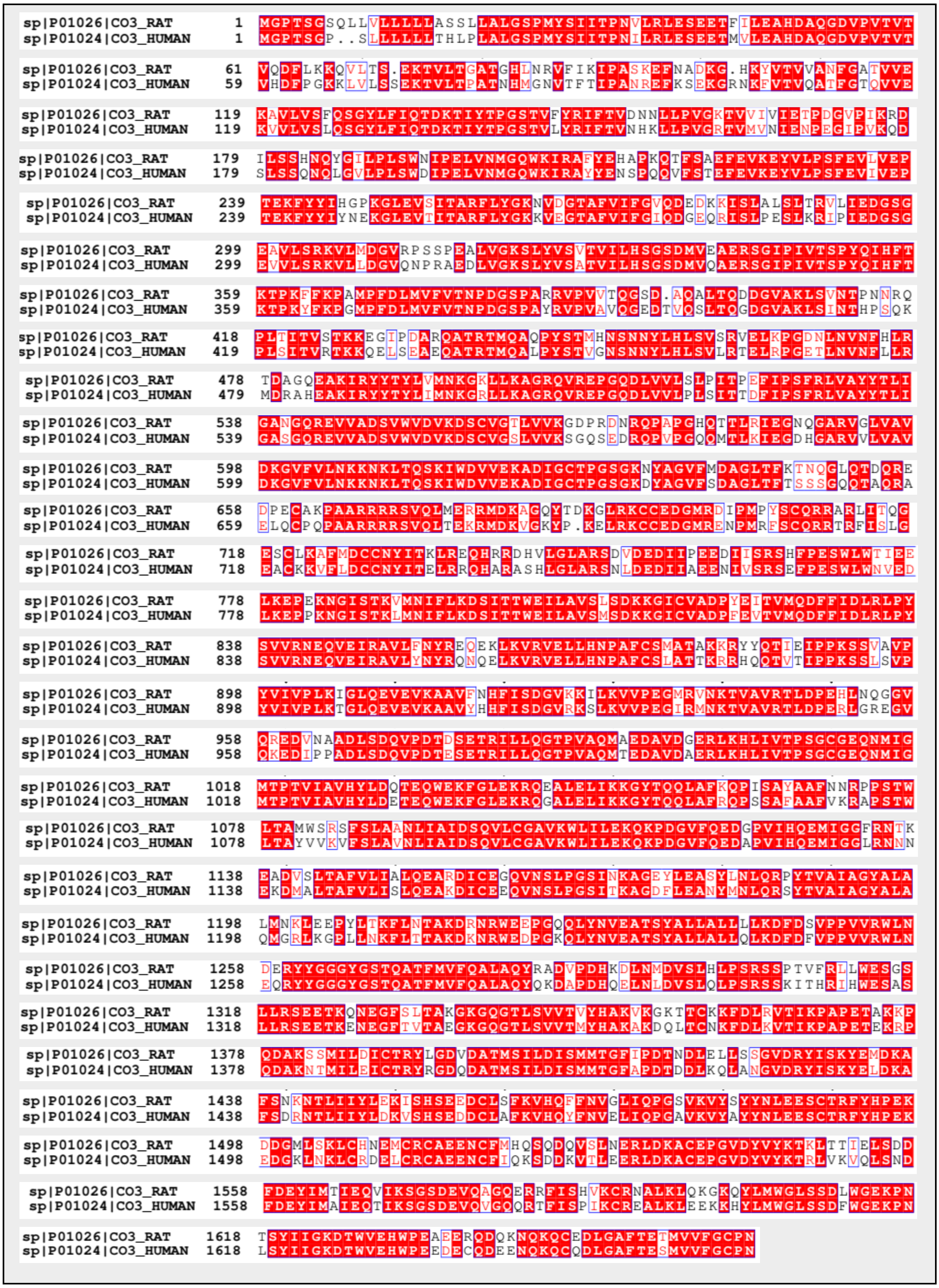

Figure XVII. Structural comparison of the complement C3 between *Rattus norvegicus* and *Homo sapiens*. The FASTA files related to rodent and human proteins were acquired on the UNIPROT search platform (http://www.uniprot.org).

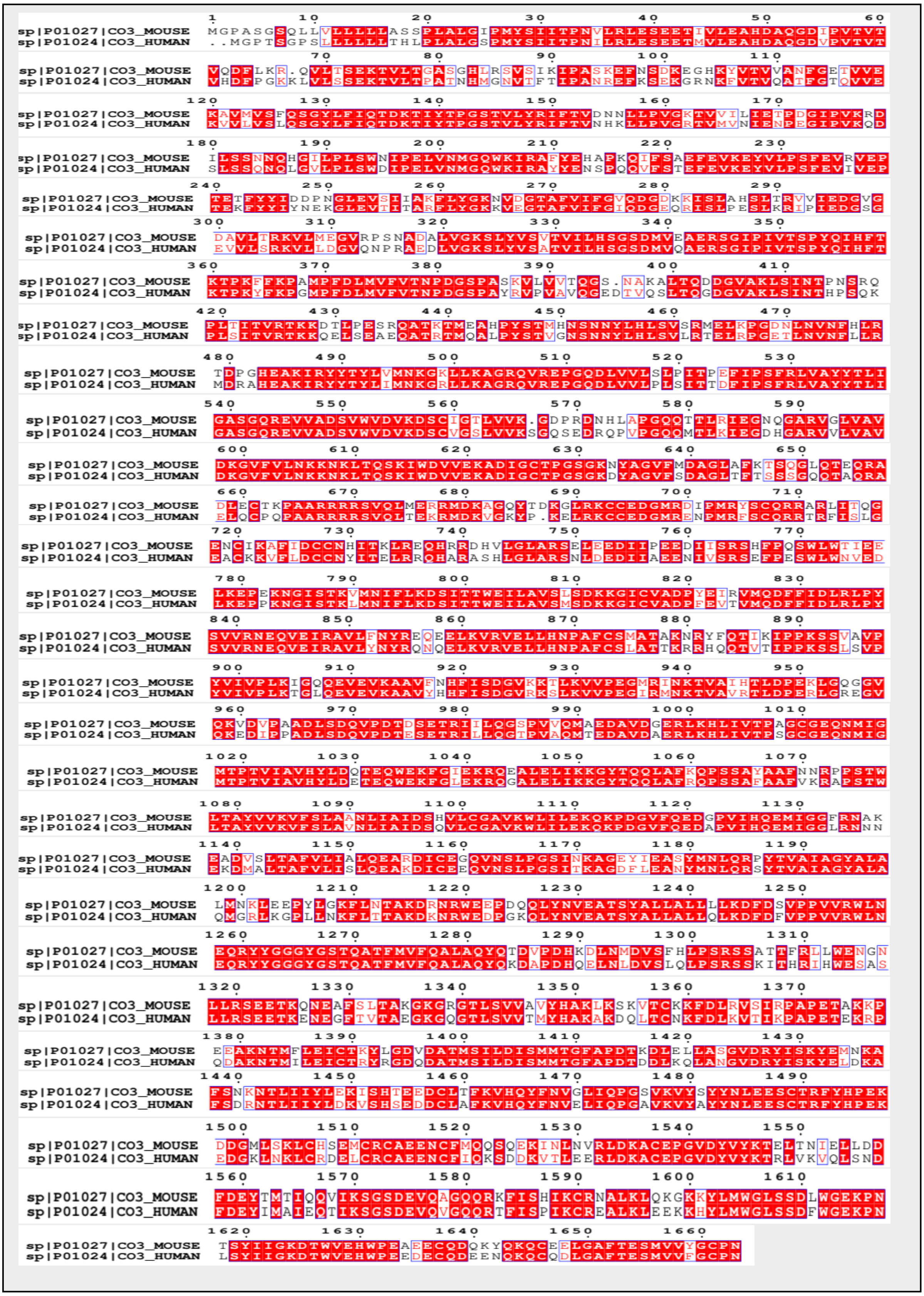

Figure XVIII. Structural comparison of the complement C3 between *Mus musculus* and *Homo sapiens*. The FASTA files related to rodent and human proteins were acquired on the UNIPROT search platform (http://www.uniprot.org).

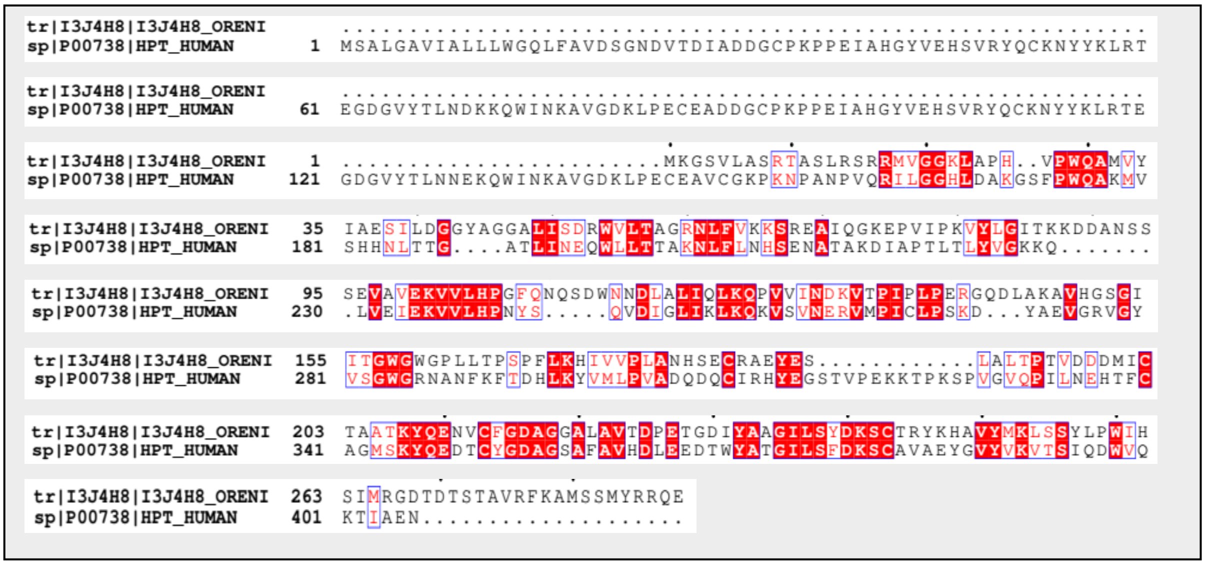

Figure XIX. Structural comparison of the haptoglobin between *Oreochromis niloticus* and *Homo sapiens*. The FASTA files related to tilapia and human proteins were acquired on the UNIPROT search platform (http://www.uniprot.org).

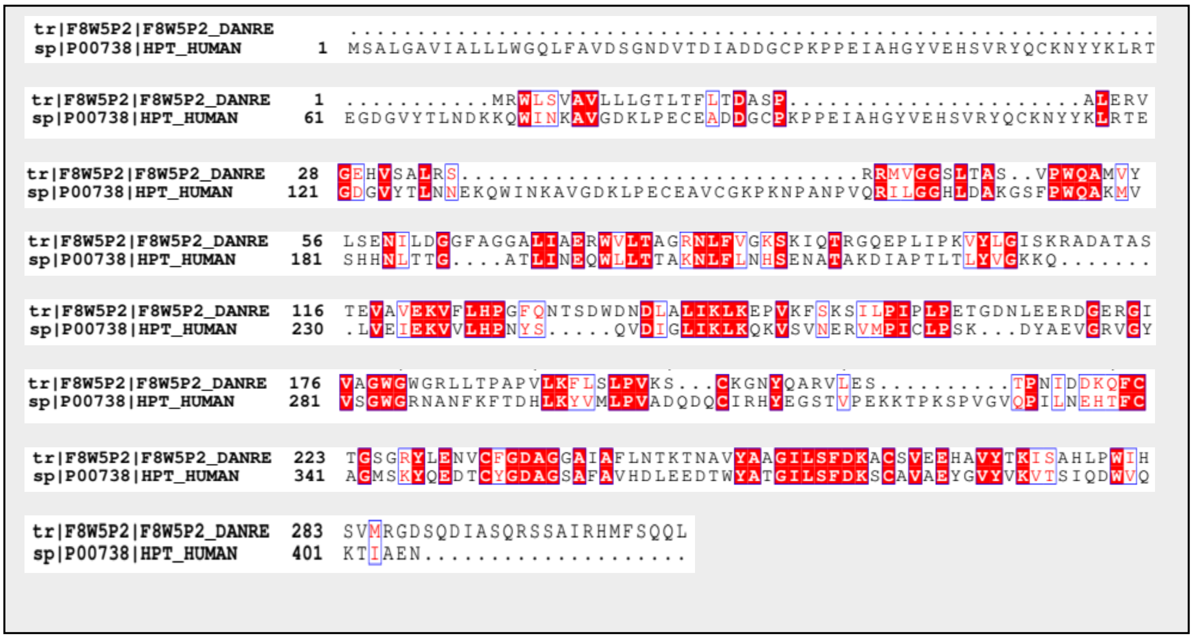

Figure XX. Structural comparison of the haptoglobin between *Danio rerio* and *Homo sapiens*. The FASTA files related to zebrafish and human proteins were acquired on the UNIPROT search platform (http://www.uniprot.org).

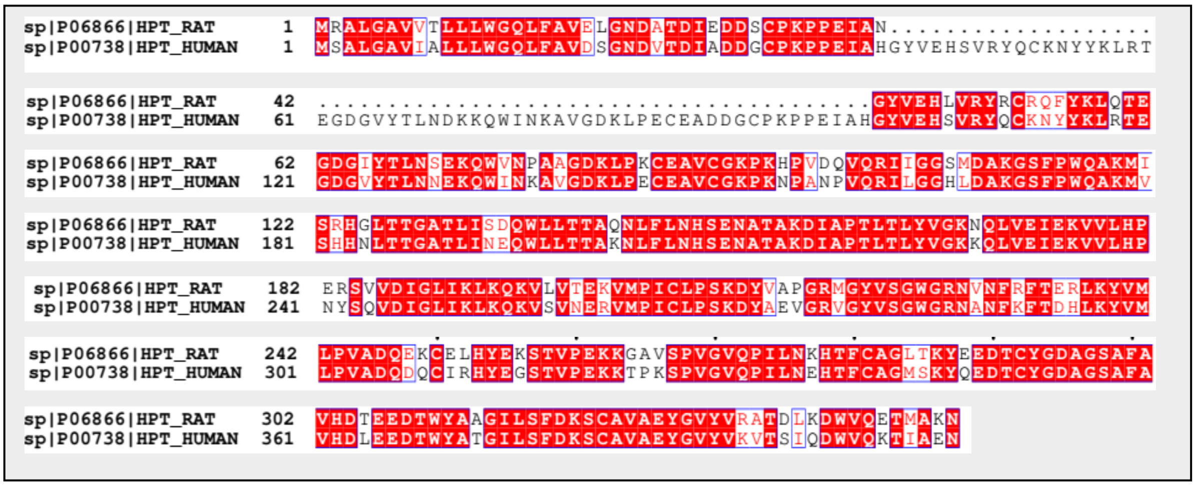

Figure XXI. Structural comparison of the haptoglobin between *Rattus norvegicus* and *Homo sapiens*. The FASTA files related to rodent and human proteins were acquired on the UNIPROT search platform (http://www.uniprot.org).

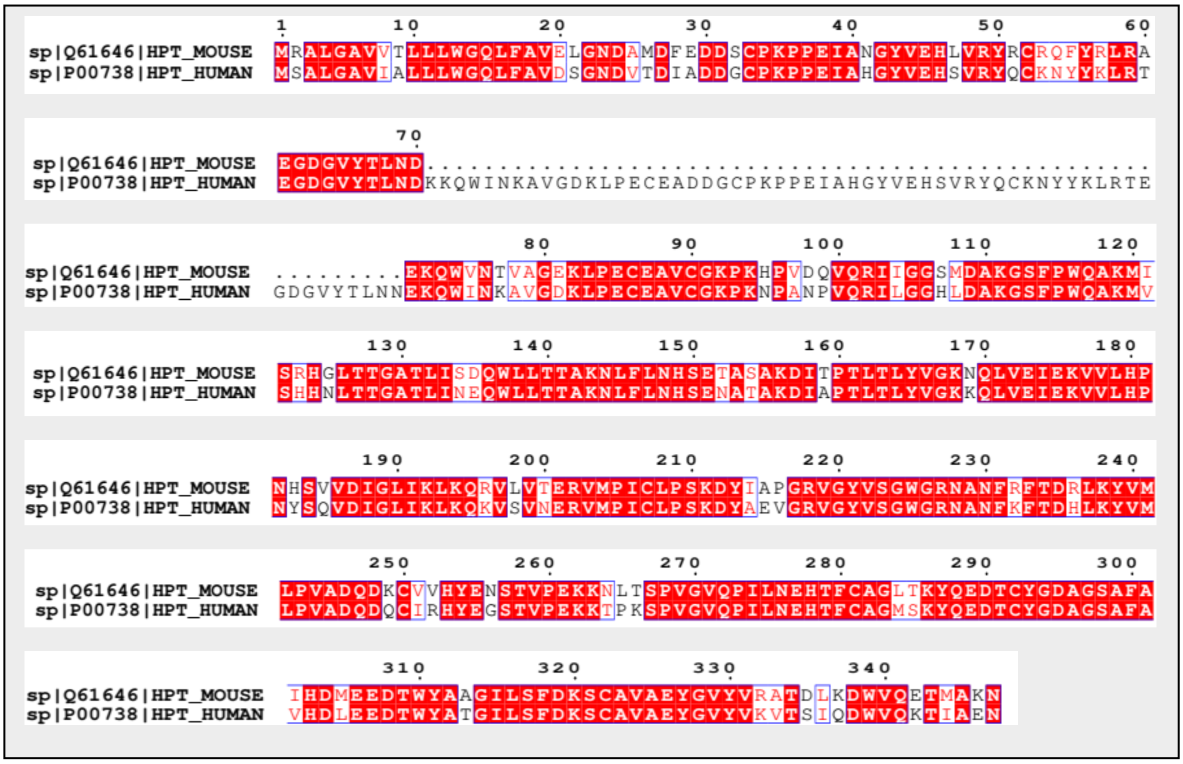

Figure XXII. Structural comparison of the haptoglobin between *Mus musculus* and *Homo sapiens*. The FASTA files related to rodent and human proteins were acquired on the UNIPROT search platform (http://www.uniprot.org).

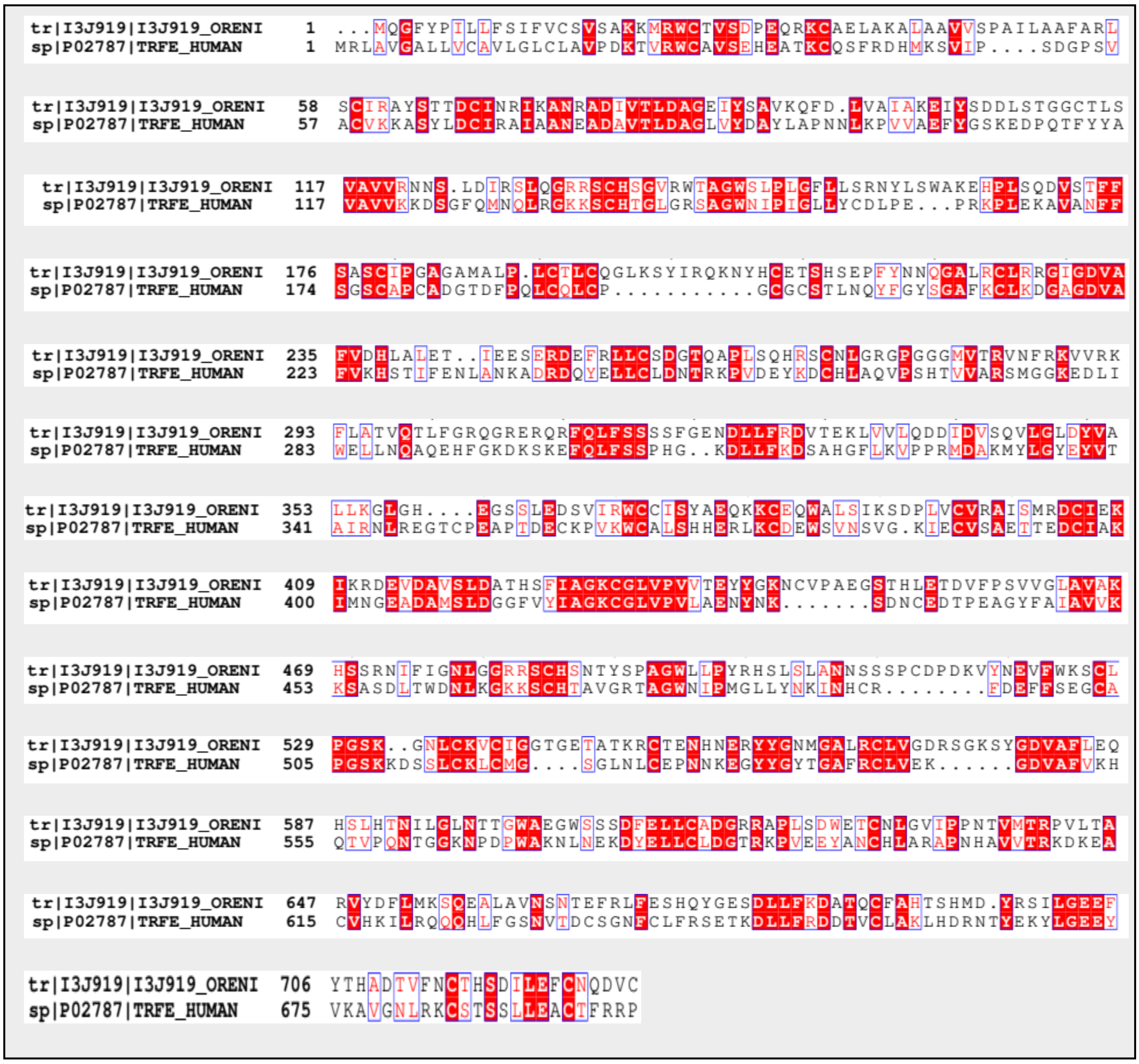

Figure XXIII. Structural comparison of the serotransferrin between *Oreochromis niloticus* and *Homo sapiens*. The FASTA files related to tilapia and human proteins were acquired on the UNIPROT search platform (http://www.uniprot.org).

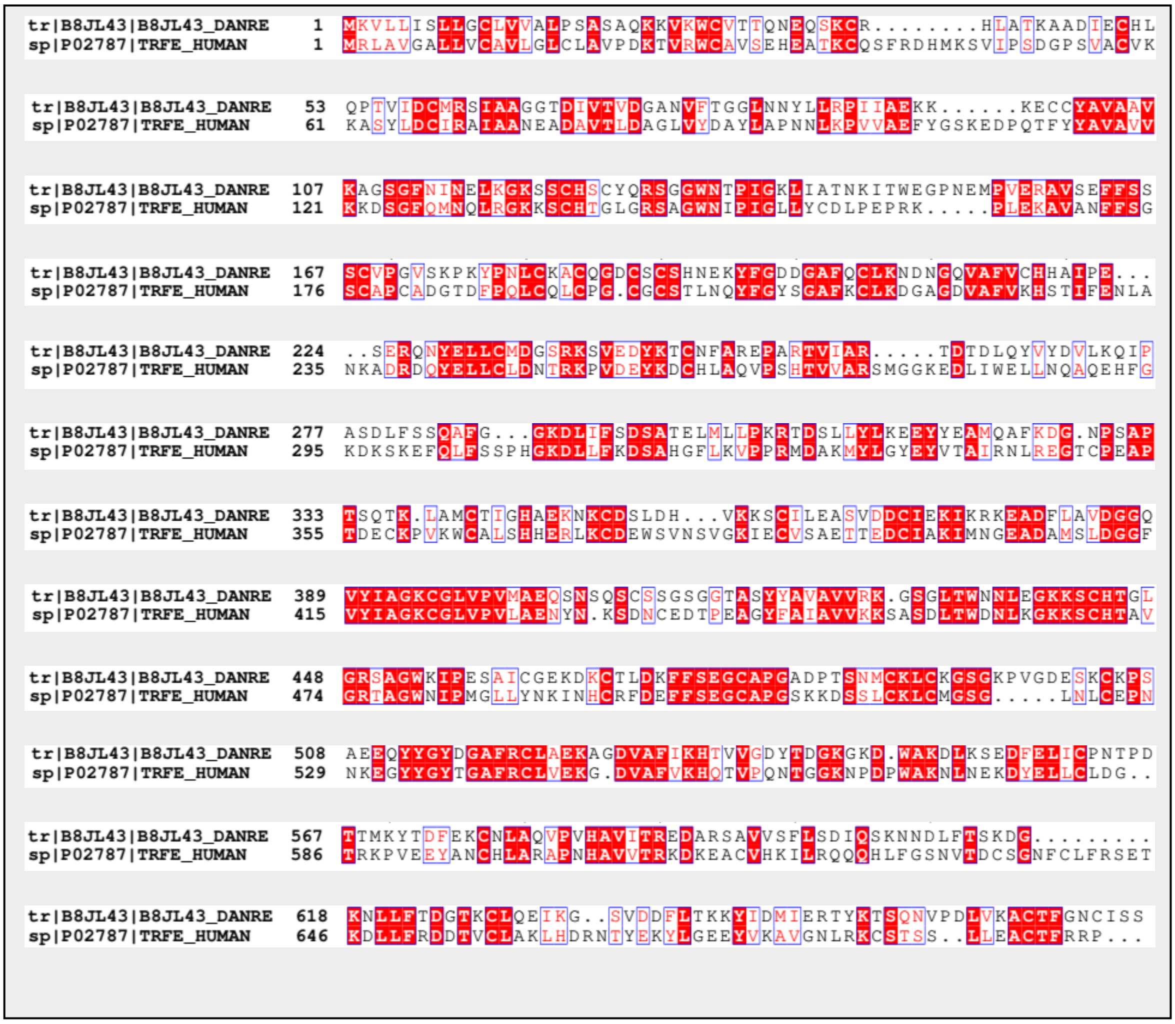

Figure XXIV. Structural comparison of the serotransferrin between *Danio rerio* and *Homo sapiens*. The FASTA files related to zebrafish and human proteins were acquired on the UNIPROT search platform (http://www.uniprot.org).

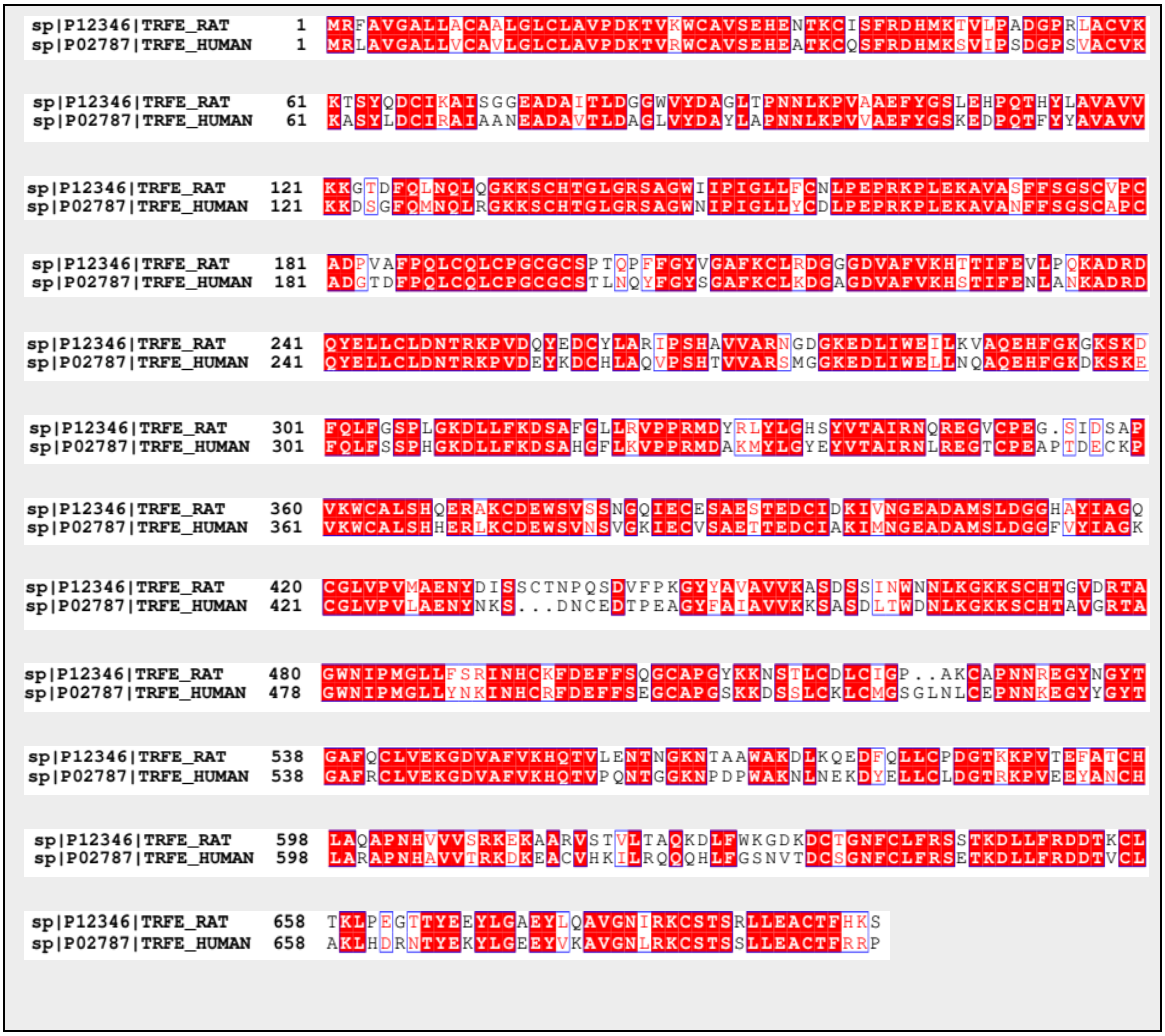

Figure XXV. Structural comparison of the serotransferrin between *Rattus norvegicus* and *Homo sapiens*. The FASTA files related to rodent and human proteins were acquired on the UNIPROT search platform (http://www.uniprot.org).

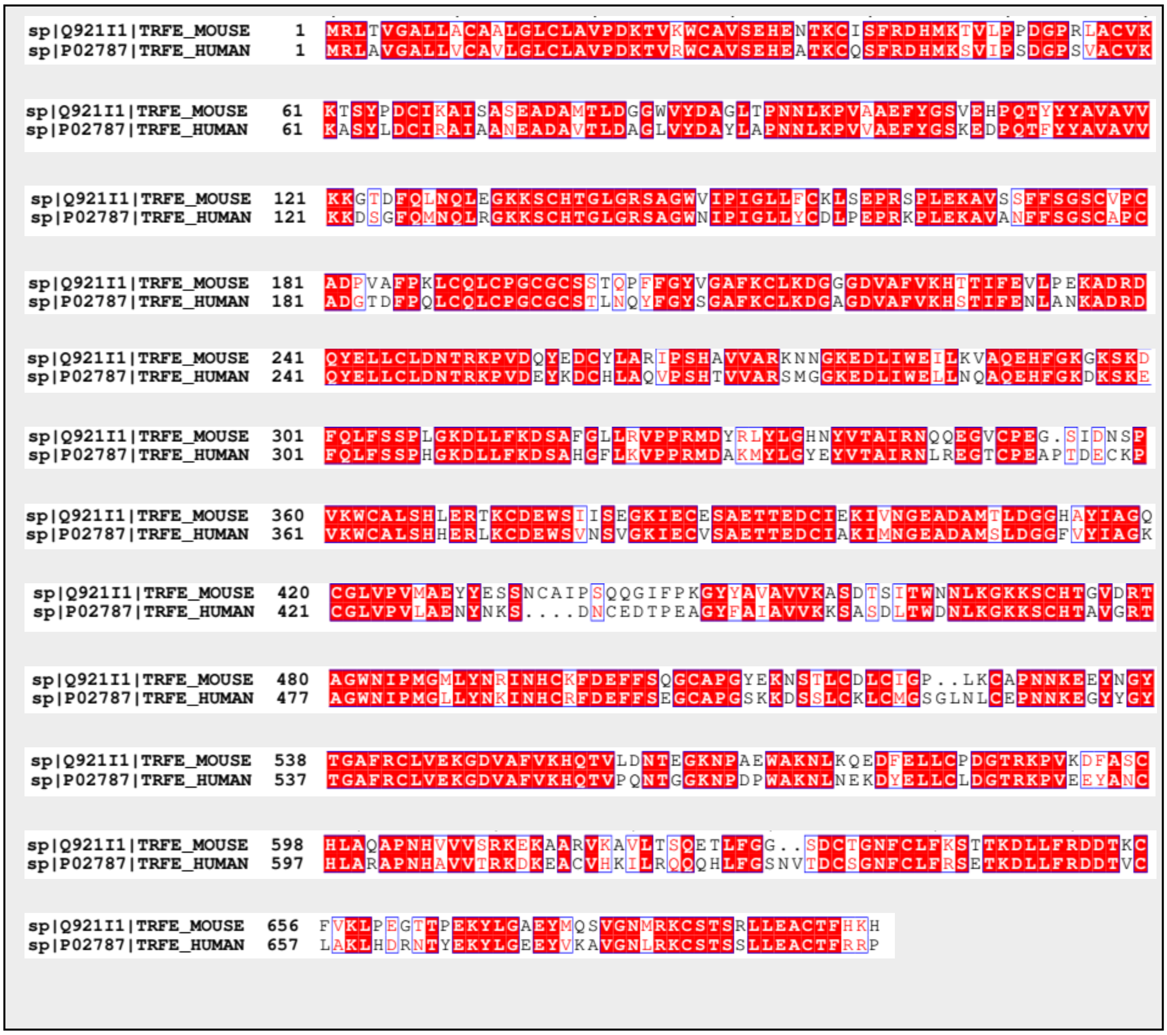

Figure XXVI. Structural comparison of the serotransferrin between *Mus musculus* and *Homo sapiens*. The FASTA files related to rodent and human proteins were acquired on the UNIPROT search platform (http://www.uniprot.org).

Attachment 3

(Blood analysis- Manual counting)

Results and discussion for blood analysis (manual counting)

Fish inoculated with *A. hydrophila* showed a significant decrease ($p < 0.05$) in erythrocyte, leukocyte and granulocyte counts 6 HPI when compared to animals inoculated with saline (Figure XXVII and XXVIII). No difference was observed in the number of circulating lymphocytes, thrombocytes and monocytes 6 and 24 HPI (Figure XXVIII). The study of hematocrit, MCV, hemoglobin and MCHC did not demonstrate significant variations during the experimental period (Figure XXVII). Similar to our results, Inoue et al.[1] showed correlation between manual counting and flow cytometry of fish blood cells. According to these authors, this technique presented advantages, because it is a simple and rapid method for analysis of fish blood cells.

Automatic counting methods have been routinely used for the diagnosis of diseases in mammals. However, the techniques applied to mammalian blood depend on lysing the erythrocytes, and then the remaining leukocytes can be counted automatically by the machine. In fish, these techniques are not precise, since their erythrocytes are nucleated, and the glutinous nature of released chromatin from red blood cells interferes with automated counting[2]. On the other hand, the limitations of the use of flow cytometry would be the high costs of the analysis, such fact difficult its use in routine clinical analysis, besides the need for technical training to carry out the sampling.

Material and Methods for blood analysis

Blood samples were drawn from the caudal vessel. Red (RBC) and white blood cell (WBC) counts were made using a haemocytometer and Natt and Herrick solution. The haemoglobin concentration (Hb) was determined with Drabkin´s reagent read at 540 nm and haematocrit (Ht) by a microhaematocrit centrifugation technique. Mean cell volume (MCV) and cell haemoglobin concentration (MCHC) were calculated from the Ht, [Hb] and RBC. Blood smears for differential leukocyte counts were stained with a combination of May-Grünwald Giemsa and Wright´s Method[3].

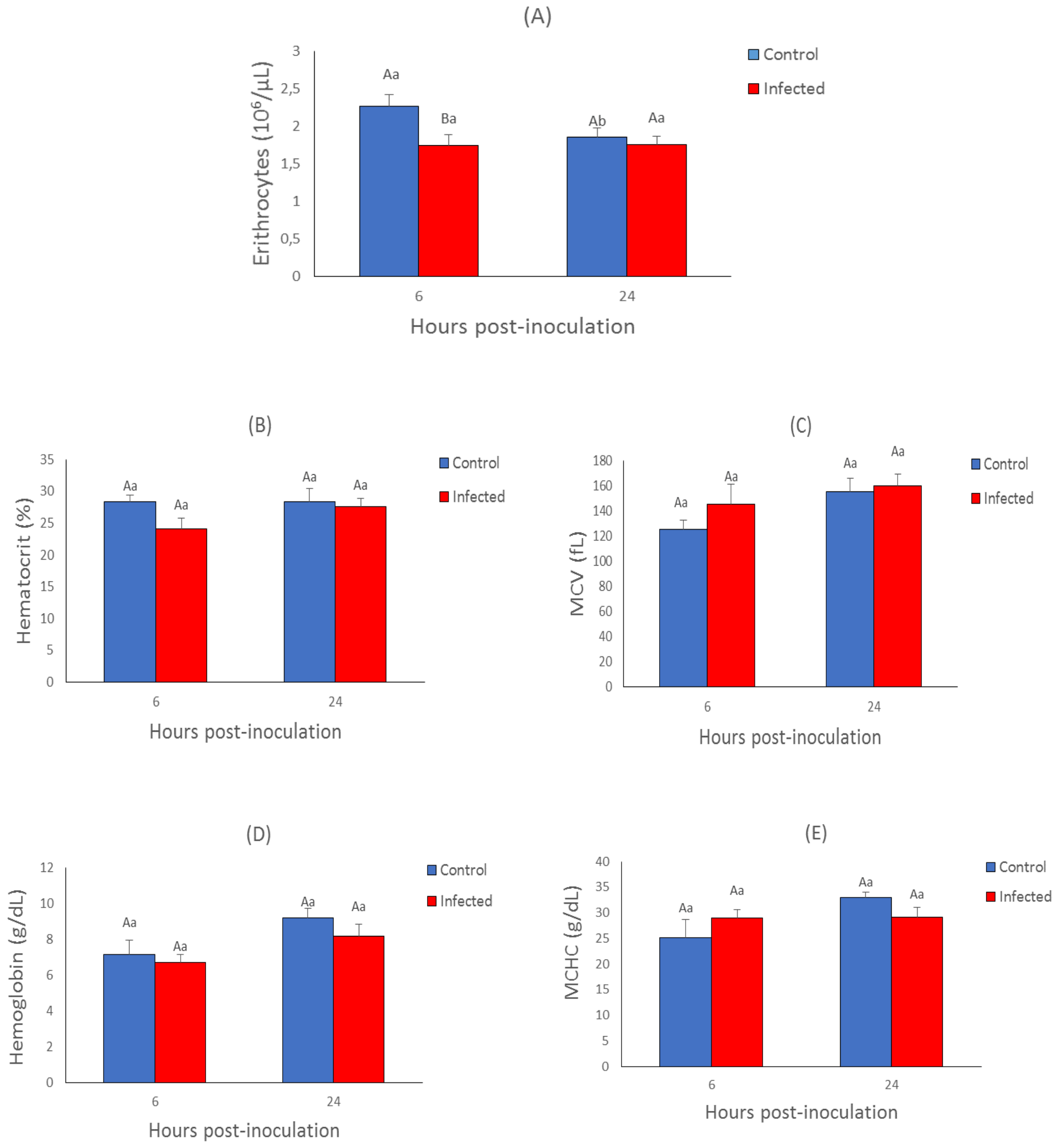

Figure XXVII. Mean values (±SE) and ANOVA for hematological parameters: (A) Number of erythrocytes; (B) Hematocrit; (C) MCV - Mean corpuscular volume; (D) Hemoglobin; (E) MCHC – Mean corpuscular hemoglobin concentration. Mean values (n=10) and ANOVA observed for total cells and differential counting of cells present in the blood during acute inflammatory response in tilapias 6 and 24 HPI. Means followed by the same letter do not differ by the Tukey test (P<0,05). The variance analysis is represented by capital letters to compare the different treatments within each experimental period, lowercase letters to compare the evolution of each treatment in the different experimental periods. Different letters indicate significant difference ($p < 0.05$).

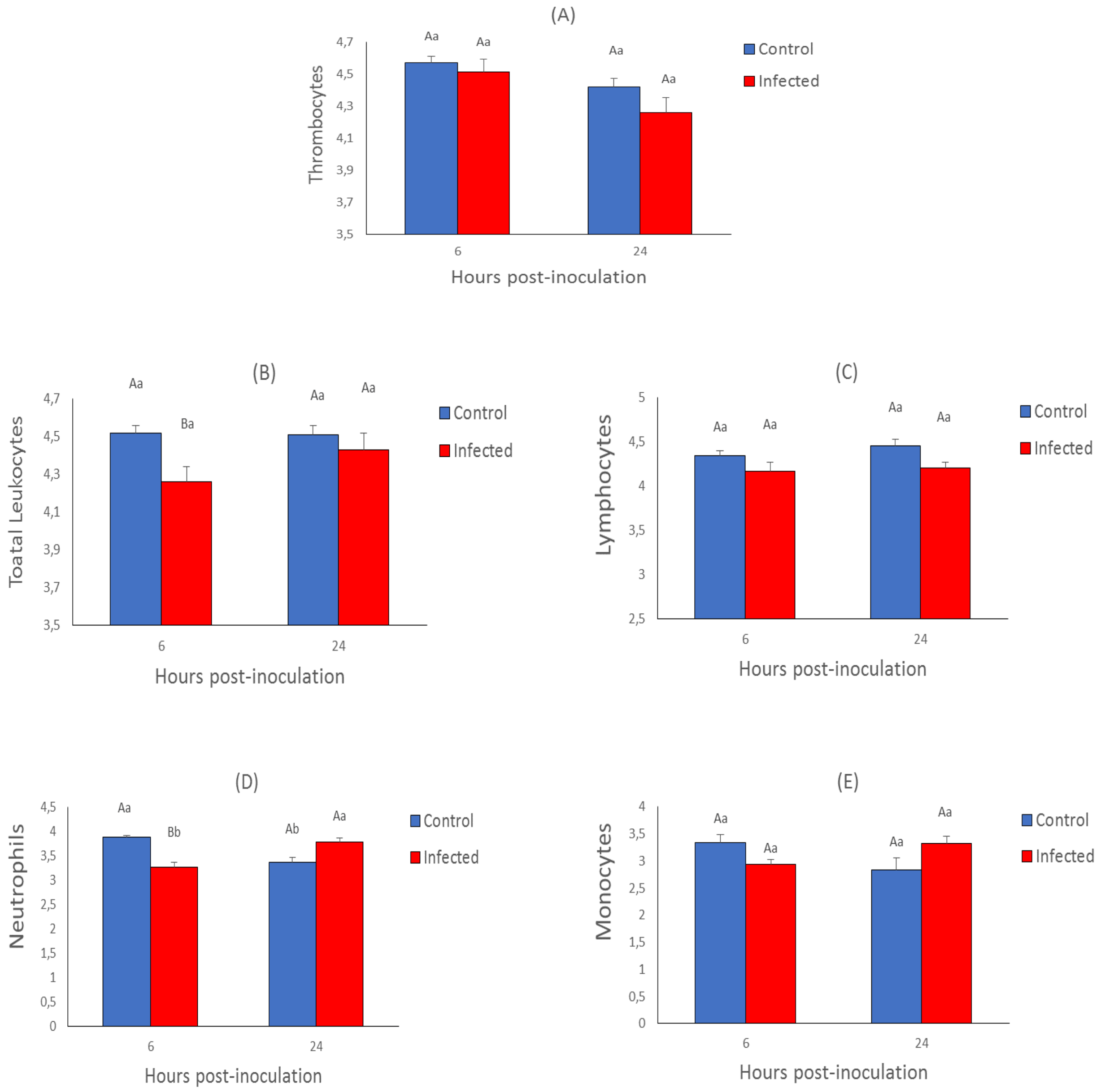

Figure XXVIII. Mean values (±SE, data transformed in Log x+1) and ANOVA for thrombocyte and leukocyte counts: (A) Number of thrombocytes; (B) Number of leukocytes; (C) Number of lymphocytes; (D) Number of neutrophils; (E) Number of monocytes. Mean values (n=10) and ANOVA observed for total cells and differential counting of cells present in the blood during acute inflammatory response in tilapias 6 and 24 HPI. Means followed by the same letter do not differ by the Tukey test ($P<0,05$). The variance analysis is represented by capital letters to compare the different treatments within each experimental period, lowercase letters to compare the evolution of each treatment in the different experimental periods. Different letters indicate significant difference ($p < 0.05$).

References

1. Inoue, T. et al. A new method for fish leucocyte counting and partial differentiation by flow cytometry. Fish Shellfish Immunol **13**, 379-390, 2002.
2. Burgess, S. C. & Davison, T. F. Counting absolute numbers of specific leukocytes subpopulations in avian whole blood using a single-step flow cytometric technique: comparison of two inbred lines of chickens. J. Immunol Methods **227**, 169–176, (1999).
3. Belo, M. A. A. et al. Haematological response of curimbas *Prochilodus lineatus*, naturally infected with *Neoechinorynchus curemai*. J Fish Biol **82** 1403-1410, (2013).
